# Robot-assisted surgery in thoracic and visceral indications: an updated systematic review

**DOI:** 10.1007/s00464-023-10670-1

**Published:** 2024-02-02

**Authors:** Nicole Grössmann-Waniek, Michaela Riegelnegg, Lucia Gassner, Claudia Wild

**Affiliations:** Austrian Institute for Health Technology Assessment (AIHTA), Garnisongasse 7/20, 1090 Vienna, Austria

**Keywords:** Robot-assisted surgery, Visceral & thoracic surgery, Laparoscopic procedures, Open surgery

## Abstract

**Background:**

In surgical advancements, robot-assisted surgery (RAS) holds several promises like shorter hospital stays, reduced complications, and improved technical capabilities over standard care. Despite extensive evidence, the actual patient benefits of RAS remain unclear. Thus, our systematic review aimed to assess the effectiveness and safety of RAS in visceral and thoracic surgery compared to laparoscopic or open surgery.

**Methods:**

We performed a systematic literature search in two databases (Medline via Ovid and The Cochrane Library) in April 2023. The search was restricted to 14 predefined thoracic and visceral procedures and randomized controlled trials (RCTs). Synthesis of data on critical outcomes followed the Grading of Recommendations, Assessment, Development, and Evaluation methodology, and the risk of bias was evaluated using the Cochrane Collaboration’s Tool Version 1.

**Results:**

For five out of 14 procedures, no evidence could be identified. A total of 20 RCTs and five follow-up publications met the inclusion criteria. Overall, most studies had either not reported or measured patient-relevant endpoints. The majority of outcomes showed comparable results between study groups. However, RAS demonstrated potential advantages in specific endpoints (e.g., blood loss), yet these findings relied on a limited number of low-quality studies. Statistically significant RAS benefits were also noted in some outcomes for certain indications—recurrence, quality of life, transfusions, and hospitalisation. Safety outcomes were improved for patients undergoing robot-assisted gastrectomy, as well as rectal and liver resection. Regarding operation time, results were contradicting.

**Conclusion:**

In summary, conclusive assertions on RAS superiority are impeded by inconsistent and insufficient low-quality evidence across various outcomes and procedures. While RAS may offer potential advantages in some surgical areas, healthcare decisions should also take into account the limited quality of evidence, financial implications, and environmental factors. Furthermore, considerations should extend to the ergonomic aspects for maintaining a healthy surgical environment.

**Graphical abstract:**

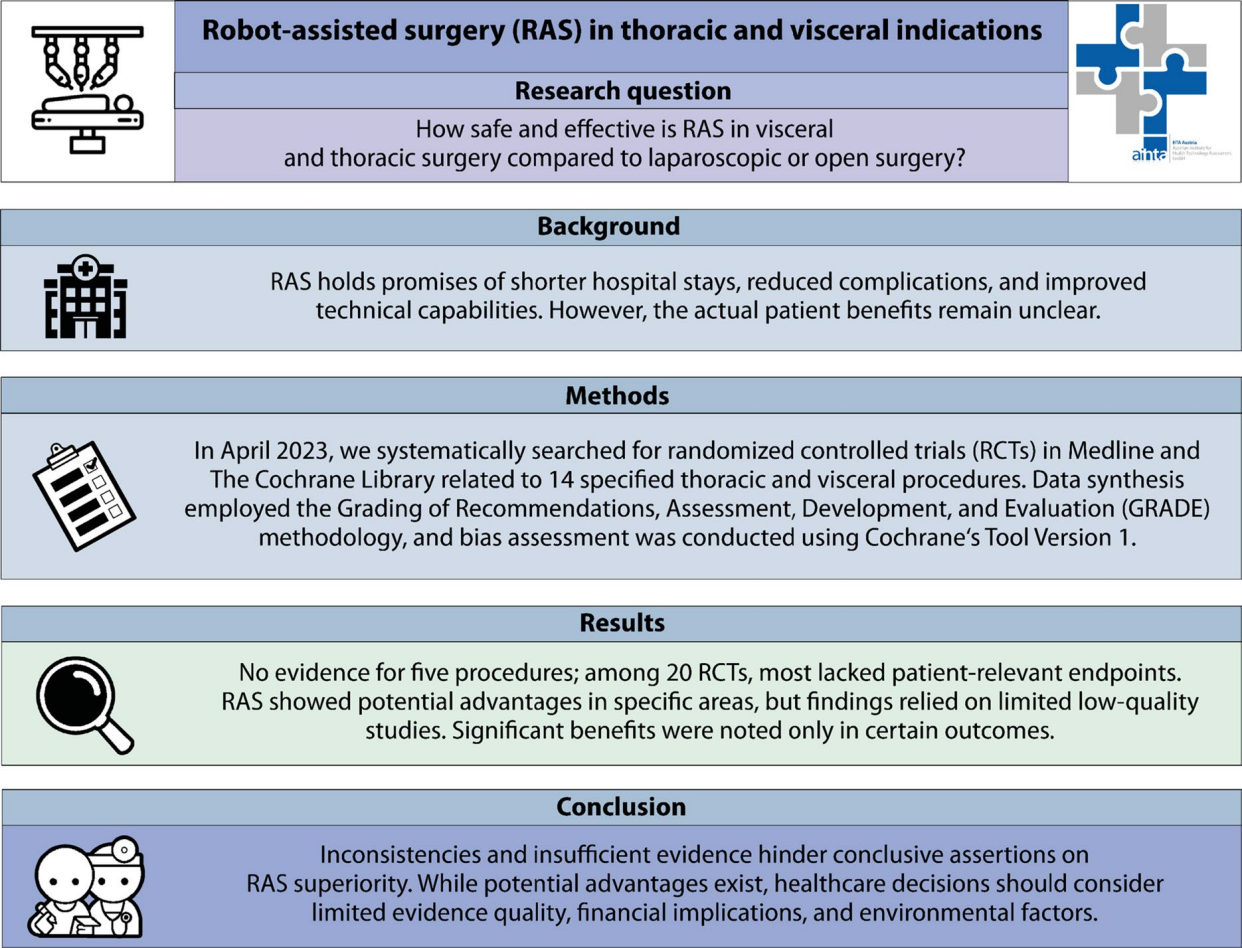

**Supplementary Information:**

The online version contains supplementary material available at 10.1007/s00464-023-10670-1.

Robot-assisted surgery (RAS) is an advanced minimally invasive procedure that is nowadays utilised in a wide clinical spectrum [1]. The robotic system’s instruments are manipulated through a direct telemanipulator, which acts as a remote device enabling the surgeon to execute typical surgical motions by controlling the robotic arms [2, 3]. Compared to conventional minimally invasive techniques, RAS is expected to offer surgical benefits in terms of visualization, dexterity, and ergonomics, while still retaining the perioperative advantages of endoscopic surgery [1, 4]. Moreover, RAS might be associated with a variety of potential advantages for patients such as a shortened hospitalisation, fastened recovery times, reduced risk of complications as well as less traumatic access into the body [5–7].

Despite anticipated benefits, RAS has not proven its superiority over conventional techniques in numerous surgical procedures [8, 9]. Additionally, the annual procedure volume of RAS has significantly increased from 136,000 in 2008 to 877,000 in 2017 [7, 10]. Hence, the European Network for Health Technology Assessment (EUnetHTA) conducted a systematic report in 2019 [11] to evaluate the effectiveness and safety of RAS in 14 thoracic and visceral indications. Notwithstanding the increasing use of RAS, a scarcity of reliable evidence was noted across almost all assessed indications, with minor improvements in clinical outcomes observed in only four procedures [11].

Currently, new technological advancements, including endoluminal robotics and the integration of artificial intelligence into robotic systems, are underway, illustrating the dynamic landscape of surgical progress [12–14]. This trend poses additional challenges in ensuring that advancements align with evidence-based practices, requiring a careful balance between embracing new technologies and ensuring their efficacy and safety through rigorous research and comparative studies [15]. The combination of these factors, along with the numerous ongoing studies identified in the 2019 report, underscores the necessity for a reassessment of the clinical effectiveness and safety of RAS. Therefore, our systematic review aims to provide an update on the evidence identified in the EUnetHTA report [11], focusing on 14 thoracic and visceral procedures in comparison to laparoscopic and open surgery.

## Materials and methods

The present updated systematic review was conducted based on the Preferred Reporting Items for Systematic Reviews and Meta-Analyses (PRISMA) statement [16] and structured according to the four domains of the Health Technology Assessment (HTA) Core Model® developed within EUnetHTA [17]. The study protocol was made publicly accessible on our institutional website (www.aihta.at) prior to conducting the systematic review.

### Visceral and thoracic procedures

#### Search strategy and selection of studies

Between April 17th and 19th, 2023, a systematic literature search in two databases (Medline via Ovid and The Cochrane Library) was conducted. The search syntax is available as a supplementary material of the review. The search was constrained to the timeframe of June 2018 to April 2023, aiming to update the existing systematic review published by EUnetHTA in 2019 [11]. The screening process was performed by two independent researchers (LG, CW). In addition, a systematic search identifying ongoing studies was conducted via the website www.clinicaltrials.gov.

#### Eligibility criteria

The included references were restricted to randomized controlled trials (RCTs) that enrolled more than 20 patients and were published in English or German. In accordance with the EUnetHTA systematic review from 2019, the search was limited to the following 14 predefined thoracic and visceral procedures [11]:

- Lung lobectomy
- Mediastinal surgery
- Anti-reflux surgery/fundoplication
- Oesophagectomy/oesophageal repair
- Heller myotomy
- Gastrectomy
- Bariatric surgery/gastric bypass
- Colectomy
- Rectal resection
- Ventral mesh rectopexy
- Small bowel resection
- Cholecystectomy
- Hernia repair
- Liver resection/hepatectomy.

#### Data extraction & methodological quality assessment

One independent researcher (MR) extracted data from eligible studies to predefined tables and another author checked the data to avoid any errors (LG). The extraction tables summarized the following attributes: study and patient characteristics, patient-relevant and safety-related outcomes as well as perioperative events and resource use. Percentages in the systematic review were rounded to an integer. Results are reported as mean ± SD unless stated otherwise.

The quality assessment of the included studies was critically appraised by two blinded authors (MR, LG) using the Cochrane Risk of Bias Tool Version 1 [18, 19]. The evidence was qualitatively synthesized, and the Grading of Recommendations Assessment, Development and Evaluation (GRADE) methodology [20] was applied to summarize the identified evidence for each critical outcome (survival, recurrence, quality of life [QoL], intra- and postoperative complications). According to GRADE strength of evidence is categorised as follows: very low, low, moderate, and high [20]. Disparities concerning the quality assessment as well as the strength of evidence were resolved through mutual consensus.

## Results

### Study selection

**Fig. 1:**
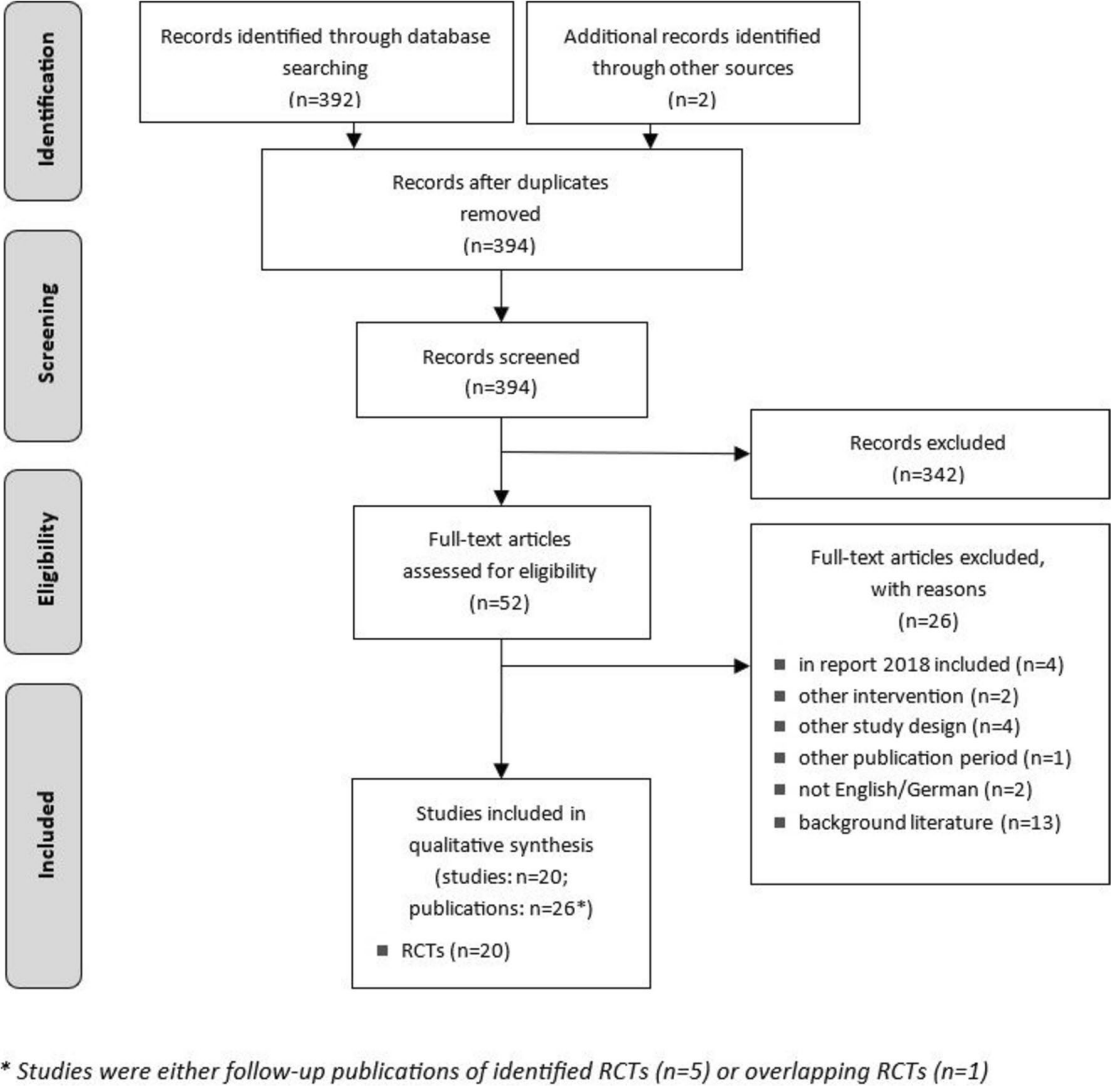
PRISMA flowchart illustrating the study selection process

In total, the systematic and manual literature search yielded 394 records after deduplication (Fig. 1). The initial screening of abstracts resulted in a total of 52 full texts that were evaluated for their eligibility. Out of these, 26 publications were selected for inclusion, comprising 20 RCTs, and six additional follow-up publications. Two of these follow-up publications are additional results of an RCT that was already included in the EUnetHTA 2019 report [11, 21, 22]. Furthermore, the results of one study were reported twice in two overlapping publications [23, 24]. To ensure comparability and transparency in the analysis, only the most recent publication was incorporated into the systematic review. Concerning five (cholecystectomy, small bowel resection, bariatric surgery/gastric bypass, Heller myotomy, mediastinal surgery) out of 14 procedures no RCTs could be identified, those will not be further discussed in the results section. Only statistically significant results and outcomes deemed critical as well as perioperative events and resource use are presented descriptively. Thus, results related to other outcomes are listed in the data extraction table in the supplementary materials (Table A1–A5).

Concerning ongoing studies, a total of 62 trials could be identified, with most of the studies investigating procedures for hernia repair (n = 17), rectal resection (n = 15), gastrectomy (n = 6) and colectomy (n = 6).

### Study characteristics

A total of 4199 patients were enrolled in 20 included RCTs, of which 2085 were part of the intervention cohorts and 2114 of the control cohorts (Table 1). The age of included patients ranged from 20 to 93 years in the intervention groups, compared to 25 to 90 years in the control groups. One RCT solely enrolled female patients [25, 26], whereas in the other 19 RCTs, the proportion of female patients varied from 8 to 74% versus 11 to 68% in the intervention and control groups, respectively. In the majority of instances, robotic-assisted procedures were compared to laparoscopic approaches. The follow-up times ranged from 7 days up to 5 years. Seven RCTs were either industry-funded or conducted by authors who were sponsored by industry. A variety of robotic systems was used; however, the most utilised ones were da Vinci Surgical Systems.

**Table 1:** Baseline study characteristics of 20 RCTs and five follow-up publications

Procedure	Indications	Interventions	Comparators	N of patients*	Age, range*	♀ pts, range in %*	Follow-up	Industry funded/COI	RCTs/FU
Thoracic surgery									
Lung lobectomy	∙ NSCLC ∙ Lung lesions	∙ Robotic-assisted lobectomy ∙ Robotic-assisted thoracoscopic surgery	∙ VA lobectomy ∙ VA thoracic surgery ∙ Thoracotomy	677 (338 vs 339)	54–78 vs 53–75	33–54 vs 29–56	90–730 d	2	4 [24, 27–29]/1 [30]
Visceral surgery									
Anti-reflux surgery	∙ Gastroesophageal reflux disease	∙ Robotic-assisted lap. fundoplication	∙ Lap. fundoplication	40 (20 vs 20)	23–71 vs 25–75	50 vs 60	12 yrs	–	1 [31]
Oesophagectomy	∙ Intrathoracic oesophageal cancer ∙ Oesophageal squamous cell carcinoma	∙ Robot-assisted minimally invasive oesophagectomy	∙ Open transthoracic ∙ Minimally invasive oesophagectomy	474 (239 vs 235)	43–75 vs 42–75	14–15 vs 15–24	3–5 yrs	1	2 [32, 33]
Gastrectomy	∙ Gastric cancer	∙ Robotic gastrectomy ∙ Robotic distal gastrectomy	∙ Open gastrectomy ∙ Lap. (distal) gastrectomy	606 (302 vs 304)	34–90 vs 40–90	33–52 vs 35–37	30–365 d	–/NR	3 [34–36]
Colectomy	∙ Cancer/benign colonic pathologies ∙ Right-sided colon cancer	∙ Robotic colectomy ∙ Robot-assisted right colectomy	∙ Lap. colectomy ∙ Lap.-assisted right colectomy	198 (78 vs 120)	20–93 vs 22–90	53–60 vs 49–54	NR/5 yrs	–	2 [37, 38]
Rectal resection	∙ Middle/low rectal cancer	∙ Robotic abdominoperineal resection ∙ Robotic surgery for rectal cancer	∙ Lap. abdominoperineal resection ∙ Conventional lap. surgery	1587 (794 vs 793)	48–70 vs 49–71	38–39 vs 35–40	3 yrs	–	2 [39, 40]
Ventral mesh rectopexy	∙ External/internal rectal prolapse	∙ Robot-assisted ventral mesh rectopexy	∙ Lap. ventral mesh rectopexy	30 (16 vs 14)	49–72 vs 56–76	100 vs 100	2–5 yrs	–	2 FUs [25, 26]
Hernia repair	∙ Ventral hernia abdominal ∙ Pelvic incisional hernia ∙ Inguinal hernia	∙ Robotic ventral hernia repair ∙ Robotic-assisted incisional hernia repair ∙ Robotic transabdominal preperitoneal repair	∙ Lap. (ventral) hernia repair ∙ Lap. incisional hernia repair ∙ Standard lap. transabdominal preperitoneal repair	465 (237 vs 228)	37–76 vs 35–72	8–74 vs 11–68	7–730 d	4	5 [41–45]/2 [46, 47]
Liver resection	∙ Synchronous colorectal liver metastases	∙ Robot-assisted lap. hepatectomy	∙ Lap. hepatectomy	122 (61 vs 61)	51–63 vs 51–64	28 vs 38	3 yrs	NR	1 [48]

*CG* control group, *COI* conflict of interest, *d* days, *IG* intervention group, *FU(s)* follow-up publication(s), *lap.* laparoscopic, *n* number, *NR* not reported, *NSCLC* non-small cell lung cancer, *RCTs* randomised controlled trials, *VA* video-assisted, *vs* versus, *yrs* years

*All data is presented in the following manner: intervention group versus control group. The following indications were excluded from the table since no evidence was available: cholecystectomy, small bowel resection, bariatric surgery/gastric bypass, Heller myotomy, mediastinal surgery

### Quality of studies and quality of evidence

The risk of bias assessment resulted in twelve out of 20 RCTs with a high risk of bias, six studies with some concerns and two with a low risk of bias. The primary factors contributing to a high risk of bias were the absence of patient blinding, selective outcome reporting, insufficient details regarding power calculations and surgeon experience as well as inadequate allocation concealment. The full risk of bias assessment is depicted in the Supplementary material Table A6. The overall quality of evidence for both clinical effectiveness and safety was low based on the GRADE assessment and can be retrieved from Table A7 in the supplementary material.

### Clinical effectiveness, safety, and perioperative events & resource use

#### Thoracic surgery

##### Lung lobectomy

*Effectiveness* Effectiveness outcomes were either not assessed, reported, or not statistically significantly different in all four RCTs and one follow-up publication [24, 27–30].

*Safety* One RCT [29] reported readmissions, showing statistically significant results favouring the intervention group (intervention group [IG]: 1 [3%] vs control group [CG]: 8 [21%]; p = 0.029).

*Perioperative events & resource use* Statistically significantly fewer cases of blood loss were observed in the intervention groups of two RCTs (median [IQR] IG: 100 [50–100] vs CG: 100 [50–150], p = 0.04 [28]; < 100 ml IG: 65 [86%] vs CG: 16 [22%], p < 0.001; ≥ 100 ml IG: 11 [15%] vs CG: 56 [78%], p < 0.001 [24]). Additionally, there were heterogeneous results concerning drain duration in three RCTs. One RCT [28] reported statistically significantly more drainage volume in the intervention group, while another RCT [24] reported this in the control group. The third RCT [29] indicated no differences.

#### Visceral surgery

##### Anti-reflux surgery

*Effectiveness & safety* Effectiveness and safety outcomes were either not assessed, reported, or not statistically significantly different in the included RCT [31].

*Safety* The identified RCT did not assess either intra- or post-operative complications [31].

*Perioperative events & resource use* The operation time was statistically significantly longer in the control group compared to the intervention cohort (IG: 88 ± 18 vs CG: 102 ± 19 min, p = 0.033) [31].

##### Oesophagectomy

*Effectiveness & safety* Effectiveness and safety outcomes were either not assessed, reported, or not statistically significantly different in both included RCTs [32, 33].

*Perioperative events & resource use* The operation time was statistically significantly shorter in the intervention group compared to the control group (IG: 203.8 ± 59.4 vs CG: 244.9 ± 61.0 min; p < 0.001) in one RCT [32].

##### Gastrectomy

*Effectiveness* None of the three included RCTs [34–36] reported any death in the study cohorts. Moreover, the outcomes recurrence and QoL were not assessed in the eligible studies.

*Safety* All three identified RCTs reported postoperative complications. Among them, two RCTs [34, 35] yielded statistically significant differences in overall morbidity (IG: 13 [9%] vs CG: 25 [18%]; p = 0.039), medical morbidity (IG: 9 [6%] vs CG: 20 [14%]; p = 0.033) [34], and in overall complications (≥ grade IIb IG: 10 (9%) vs CG: 23 (20%), p = 0.02; ≥ grade IIIa IG: 6 (5%) vs CG: 19 (16%), p = 0.01) [35] favouring the intervention group.

*Perioperative events & resource use* All three RCTs [34–36] reported instances of blood loss, which were statistically significantly lower in the intervention group in two studies (IG: 41.2 ± 45.7 vs CG: 55.7 ± 70.5, p = 0.045 [34]; IG: 123.7 ± 89.3 vs CG: 276.3 ± 152.1, p < 0.001 [36]). Operation time was documented in all three studies, revealing statistically significantly longer operation times in the intervention groups of two RCTs (IG: 297 [179–654] vs. CG: 245 [131–534] min, p = 0.001 [35]; IG: 353.8 ± 96.4 vs. CG: 214.6 ± 41.6 min, p < 0.001 [36]).

##### Colectomy

*Effectiveness & safety* Effectiveness and safety outcomes were either not assessed, reported, or not statistically significantly different in both included RCTs [37, 38].

*Perioperative events & resource use* The two included studies reported statistically significant results favouring the control arms regarding the duration of surgery (IG: 195 ± 41.0 vs CG: 129.7 ± 43.2 min, p < 0.001 [37]; median [range] IG: 172 [107–353] vs CG: 145 [69–380] min, p = 0.005 [38]).

##### Rectal resection

*Effectiveness* Effectiveness outcomes were either not assessed, reported, or not statistically significantly different in both included RCTs [39, 40].

*Safety* In one of the two eligible studies [40] statistically significant differences were reported in favour of the robotic-assisted study group concerning intraoperative events (IG: 32 [6%] vs CG: 51 [9%], p = 0.030). Both RCTs [39, 40], showed statistically significantly fewer postoperative complications of Clavien–Dindo grade II or higher in the robotic-assisted group compared to the laparoscopic group (IG: 23 [13%] vs CG: 41 [24%], p = 0.013 [39]; IG: 95 [16%] vs CG: 135 [23%], p = 0.003 [40]).

*Perioperative events & resource use* RAS was linked to statistically significantly less blood loss compared to laparoscopic surgery in both RCTs (p < 0.001 [39]; p < 0.0001 [40]). In one RCT [39], the intervention group experienced statistically significantly longer operation times compared to the control arm (median [IQR] IG: 205 [195–220] vs CG: 195 [160–238] minutes; p = 0.004). Both studies demonstrated statistically significantly shorter hospital stays for patients undergoing robotic-assisted surgery (p < 0.001 [39]; p = 0.0001 [40]).

##### Ventral mesh rectopexy

*Effectiveness & safety, perioperative events & resource use* Effectiveness and safety outcomes as well as perioperative events and resource use were either not assessed, reported, or not statistically significantly different in the two included follow-up studies [25, 26].

##### Hernia repair

*Effectiveness* None of the included RCTs [41–45] and follow-up publications [46, 47] assessed survival outcomes. After 1 year of surgery, statistically significantly more recurrences occurred in the intervention group as reported in a follow-up publication [46] (clinical recurrence IG: 5/20 [25%] vs CG: 0/17 [not reported]; 37/75 [49%], p = 0.03; composite recurrence IG: 9/38 [24%] vs CG: 2/33 [6%]; 71/75 [95%], p = 0.04). In the same follow-up study [46], a statistically significant improvement in hernia-specific QoL assessment was observed 1 year after surgery in the intervention group compared to the laparoscopic counterparts (p = 0.04).

*Safety* None of the eligible studies identified statistically significant differences regarding intra- and postoperative complications.

*Perioperative events & resource use* The operation time, measured in minutes, was statistically significantly longer in the intervention groups of four RCTs:

- IG: 146 (IQR: 123–192) vs CG: 94 (IQR: 69–116) (p < 0.001) [41]
- IG: 355.6 ± 89 vs CG: 293.5 ± 89 (p = 0.04) [42]
- time from skin incision to closure (median [IQR] IG: 75.5 [59.0–93.8] vs CG: 40.5 [29.2–63.8], p < 0.001), time for dissection of the hernia (IG: 18.0 [12.0–27.0] vs CG: 13.0 [7.0–23.0], p = 0.012), time for mesh fixation (IG: 6.88 [5.00–9.00] vs CG: 1.00 [NR]; p < 0.001) and time for peritoneal closure (IG: 7.00 [5.00–9.00] vs CG: 2.00 [1.00–3.00], p < 0.001) [44]
- IG: 141 ± 56 vs CG: 77 ± 37, relative rate (95% CI) 62.89 (45.75–80.01) (p < 0.001) [45].

##### Liver resection (hepatectomy)

*Effectiveness* Effectiveness outcomes were either not assessed, reported, or not statistically significantly different in the included RCT [48].

*Safety* Statistically significant differences between robotic-assisted and laparoscopic hepatectomy were observed in terms of total complications (IG: 2 [3%] vs CG: 8 [13%], p = 0.048) [48].

*Perioperative events & resource use* Statistically significant increases in blood loss were observed in the control group of the identified RCT [48] (IG: 203.11 ± 10.98 vs CG: 356.00 ± 32.00 millilitres; p < 0.001). Laparoscopic surgery took statistically significantly longer than robotic-assisted laparoscopy (Intervention Group: 156.34 ± 15.97 vs Control Group: 184.18 ± 18.03 min, p < 0.001), as reported in the single identified RCT [48]. Additionally, statistically significantly more transfusions were necessary in the control group of the eligible study [48] (IG: 608.31 ± 117.08 vs CG: 656.21 ± 103.75, p = 0.018).

## Discussion

The rapid introduction of expensive technological advances poses a challenge for policymakers as it often surpasses the capacity of governments and society to promptly adapt to the resulting changes, leading to heightened inequalities and ethical dilemmas [15, 49]. Thus, the integration of new technologies frequently precedes the accumulation of solid evidence demonstrating clear superiority [15]. This phenomenon is exemplified by the fast diffusion of robot-assisted prostatectomy in Europe, demonstrating a substantial increase in performed procedures from 3% in 2008 (Germany) to 46% in 2018, highlighting the early adoption of advanced surgical techniques without an initial robust evidence base [50].

Nowadays RAS is introduced into a variety of surgical specialities with immense evidence generated solely on Intuitive-Surgical products of 13,500 peer-reviewed articles, averaging one publication every 4 h [51]. Despite this huge evidence base and the broad application, it is still unknown if RAS overcomes the limitations of conventional approaches [51–53]. Therefore, we aimed to systematically analyse the effectiveness and safety of RAS in 14 thoracic and visceral indications compared to laparoscopic or open surgery. For nine indications of interest, a total of 20 RCTs and five follow-up publications could be identified. In general, the overall quality of evidence was low with more than half of the studies (n = 12) exhibiting a high risk of bias.

In summary, among the investigated study endpoints, rectal and liver resections were the only indications that demonstrated advantages with RAS over conventional procedures in at least four outcomes of interest. Notwithstanding claims of superiority, statistically significant differences favouring RAS were solely observed in one respective indication across several investigated outcomes—recurrence, QoL, transfusions, and hospitalization (Table 2). Notably, safety outcomes were enhanced for patients undergoing robot-assisted gastrectomy, as well as rectal and liver resection. Moreover, operation time yielded conflicting results; shortened surgery times were observed exclusively in robot-assisted liver resections and fundoplications, while five indications showed prolonged surgeries. Additionally, reduced blood loss was observed in the intervention groups of five indications: lung lobectomy, oesophagectomy, rectal resection, liver resection, and gastrectomy. Overall, we could see that in a great deal of instances, patient-relevant outcomes were not available or comparable to the results of the control standard intervention.

**Table 2:**
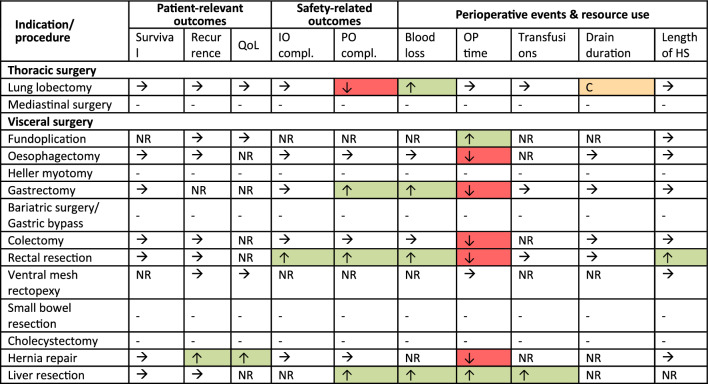
Overview of study results of included thoracic and visceral procedures (Color figure online)

*C and orange colour* conflicting evidence, *compl.* complications, *G.* Gallbladder, *HS* hospital stay, *IO* intraoperative, *NR* the study outcome was not reported, *OR* original report, *PO* postoperative, *sat.* satisfaction, *U* update report, *↑ and green colour* at least one study reported statistically significant results favouring the intervention group, *↓ and red colour* at least one study reported statistically significant results favouring the control group, **→** no study reported statistically significant results, – no study was identified

Our results align with the EUnetHTA report published in 2019 [11], serving as the basis for our updated systematic review. Schmid et al. noted eligible evidence for potential advantages of RAS in only four procedures. However, for the remaining five procedures for which our systematic search yielded no study updates, the EUnetHTA report identified a sole potential benefit in one outcome of interest [11]. Additionally, a 2021 systematic review of reviews from Muaddi et al. [52] also emphasized the lack of substantial supporting evidence, mainly demonstrating comparable outcomes between RAS and respective comparators. Yet, a discrepancy arises concerning robotic rectal surgery, as the 2021 systematic review [52] failed to observe any advantages, contrary to our findings where RAS approaches for rectal indications appear more promising.

Our systematic review has certain limitations, in particular the substantial heterogeneity of results stemming from diverse indications and outcomes. This heterogeneity makes comparisons and analyses challenging, thereby hindering the creation of a meta-analysis. In line with the EUnetHTA report, we exclusively included RCTs, potentially missing out on good-quality prospective non-randomized trials. However, the study from Muaddi et al. [52] from 2021 identified numerous non-randomized observational studies, both retrospective and prospective, which were mainly constrained by biases such as residual confounding, selection bias, and observer bias. Finally, we did not address any cost aspects of RAS, which would be particularly important, especially in the purchasing decision of health care systems.

Optimal future RCTs on RAS should be independent, well-powered, and ideally unbiased, incorporating patient-relevant outcomes. In addition, it is crucial to include surgeon-related outcomes, such as determining the optimal case volume required to maintain training and expertise in RAS, as well as assessing ergonomic aspects. These outcomes are notably relevant but were lacking in our analyses. Surgeon case volume is especially relevant since higher case numbers not only enhance surgeons’ skills but also contribute to the overall cost-effectiveness of RAS [54, 55]. Generally, ergonomic considerations involve optimizing the design and utilization of robotic systems to enhance surgeons’ comfort, efficiency, and safety during operations [56]. Thus, future studies should prioritize investigating ergonomic aspects to understand how design features and surgeon interactions influence performance and outcomes such as fatigue, stress levels, and procedural efficiency potentially leading to guidelines for a healthier surgical environment [57]. Additionally, studying ergonomic implications could contribute to standardised training protocols and guidelines that are currently lacking.

Besides the ongoing debate on consistent training programs for RAS surgical teams and the vast increase in the number of RAS procedures, there are growing concerns about healthcare sustainability associated with RAS [10, 55, 58, 59]. A study from Gkegkes et al. [54] concluded that factors such as a high volume of cases, competitive industry presence, and utilization of a multidisciplinary team can contribute to making the RAS more reasonable and cost-effective. Likewise, the duration of surgery is another crucial factor that can lead to significant fluctuations in the overall surgical expenses especially for public health systems [54]. However, our systematic review demonstrates that there are great variations concerning operation times with RAS, since studies have shown both reduced as well as prolonged surgery duration. Thus, there is no general statement possible, if RAS leads to improvements in the time of surgery and therefore, reduces surgical costs.

In addition to increased costs associated with RAS, healthcare decision-making should also factor in environmental sustainability. Thus, a systematic review conducted in 2022 [60] suggests that the increased environmental impact associated with RAS, as opposed to conventional laparoscopic procedures, may not adequately offset the potential clinical benefits. Factors contributing to the elevated environmental impact include higher levels of greenhouse gas emissions (44%) and waste production (24%), as well as a lower reduction in disability-adjusted life years per ton of carbon dioxide and waste [60]. These findings align with another study conducted by Woods et al. [61], which also demonstrated a 38% increase in the total carbon footprint associated with robot-assisted laparoscopy compared to conventional laparoscopy procedures.

In summary, due to the lack of consistent and sufficient high-quality evidence across various outcomes and procedures, making a conclusive statement regarding the superiority of RAS is challenging. While RAS may show promise for specific indications, the limited quality of evidence, along with financial and environmental considerations, must be weighed in purchasing decisions. Moreover, surgical societies can enhance treatment outcomes, improve health benefits for operating surgeons, and promote cost-effectiveness by implementing thorough training programs [56, 62].

### Supplementary Information

Below is the link to the electronic supplementary material.

Supplementary file1 (DOCX 116 kb)

Supplementary file2 (DOCX 30 kb)

Supplementary file3 (DOCX 170 kb)

Supplementary file4 (DOCX 31 kb)
