# Supplementary material for: Robot-assisted surgery in thoracic and visceral indications: an updated systematic review
Source: Surg Endosc. 2024 Feb 2;38(3):1139–50. doi: 10.1007/s00464-023-10670-1 (PMC10881599; doi:10.1007/s00464-023-10670-1)
Supplement: Supplementary file 1 — Supplementary file1 (DOCX 116 kb) [file 464_2023_10670_MOESM1_ESM.docx]

Extractiontables

*Table A1: Extraction tables Lung Lobectomy*

| **Lung Lobectomy** | | | |
| --- | --- | --- | --- |
| **Author, year** | **Jin et al. 2022 & 2023** | | **Veronesi et al. 2021** |
| **Study characteristics** | | | |
| Study design, indication | Single-centre, open-labelled, parallel-arm, noninferiority RCT of patients with NSCLC | | Prospective, randomised, multi-centre study of patients with NSCLC |
| Country | China | | Italy, USA |
| Funding/Sponsor | National Natural Science Foundation of China (81871882, 82072557), Robotic Research Grant from Intuitive Surgical, Inc, Shanghai Municipal Education Commission-Gaofeng Clinical Medicine Grant Support (20172005), and Outstanding Academic Leader of Shanghai (20XD1402300). | | Umberto Veronesi Foundation (Milan, Italy) and Intuitive Surgical Inc. (Sunnyvale, CA, USA) |
| Intervention (IG) \| Product | Robotic-assisted lobectomy \| da Vinci S/Si | | Robotic-assisted lobectomy \| da Vinci Robotic System |
| Comparator (CG) | Video-assisted lobectomy | | Video-assisted thoracic surgery |
| Experience of surgeon(s); time period | Surgeries were performed by the same surgical group headed by 1 experienced surgeon (>100 procedures by each approach before initiation of this study);  May 2017- May 2020 (randomisation) | | >30 major lung resections performed using one or each of the two techniques;  April 2017 to November 2018 (eligibility screening) |
| Number of randomised patients | 363; IG: 181; CG: 182 (2022) | | 77; IG: 38; CG: 39 |
| Inclusion criteria | - 18-80 yrs - Satisfactory preoperative laboratory testing - Adequate pulmonary function - ASA Score of I to III | | - >18 yrs - Known or suspected NSCLC (In case of suspected lung cancer with no preoperative diagnosis, frozen section was indicated during surgery to confirm the disease. If a benign lesion was diagnosed, the patient was considered a dropout of the study.) - pts in clinical stage T1–T2–T3, N0–N1, candidate for lobectomy, anatomical segmentectomy, or bilobectomy - pts with multiple lung tumours could be included if they could be resected with a lobectomy, lobectomy plus segmentectomy, or bilobectomy and each tumour should be staged separately - ASA Score 1-3 |
| Primary/secondary endpoints | - Primary: 3-yrs overall survival rate, extent of LN dissection - Secondary: 3-y disease-free survival, R0 resection rate, duration of surgery, intraoperative blood loss, the conversion rate, postoperative hospital stay, the incidence of postoperative adverse events, and medical costs | | - Primary: rate of conversions, bleeding, and perioperative complications (assessed by modified Clavien-Dindo scale) - Secondary: duration of surgery, number of resected LNs, number of dissected LN stations, postoperative hospital stay, postoperative pain with daily evaluation, quality of life by EORTC QoL-C30, postoperative respiratory function, and rate of local or distant recurrence at 2 yrs |
| Follow-up (months) | Every 6 months until the patient death or the completion of the study | | 2 yrs |
| Dropouts (n (%)) | 43 before or during surgery  IG: 24 (13.3); CG: 19 (10.4) (2022)  IG: 50 (27.6); CG: 48 (26.4)^^[[1]](#footnote-1)^^ at 48-wk follow-up (2023) | | After the intention-to-treat analysis  IG: 3; CG: 2 |
| **Patient characteristics** | | | |
| Age of patients (yrs., mean) | *median (IQR);* p=NS  IG: 61 (54-66); CG: 62 (53-68) | | *mean±SD; p-value*; p=NS  IG: 69±8.7; CG: 68±7.4^^[[2]](#footnote-2)^^ |
| Sex (% female) | IG: 48.4; CG: 53.4; NS | | IG: 43; CG: 41; NS |
| BMI (kg/m², mean) | *median (IQR)*; **p=0.05**  IG: 23.4 (21.7-25.6); CG: 22.9 (21.4-24.4) | | *mean±SD; p-value*  IG: 27±4.1; CG: 26±4.2; NS |
| Clinical classification | T Stage (IG vs CG; n (%); p=NS  I: 137 (87.3) vs 141 (86.5)  II: 17 (10.8) vs 20 (12.3)  III: 1 (0.6) vs 1 (6.1)  IV: 2 (1.3) vs 1 (6.1)  N Stage (IG vs CG; n (%); p=NS  0: 138 (87.9) vs 146 (89.6)  I: 8 (5.1) vs 6 (3.7)  II: 11 (7.0) vs 11 (6.7)  TNM Stage (IG vs CG; n (%); p=NS  Ia: 123 (78.3) vs 127 (77.9)  Ib: 11 (7.0) vs 12 (7.4)  IIa: 1 (0.6) vs 5 (3.1)  IIb: 9 (5.7) vs 7 (4.3)  IIIa: 13 (8.3) vs 12 (7.4) | | ASA Score (IG vs CG; n (%); p=NS  I-II: 18 (56) vs 24 (65)  III: 14 (44) vs 13 (35)  Clinical Stage (IG vs CG; n (%); p=NS  Ia: 27 (77) vs 25 (71)  Ib: 6 (17) vs 7 (20)  IIa: 2 (6) vs 1 (3)  IIb: 0 (0) vs 2 (6) |
| **Patient-relevant outcomes** | | | |
| Survival (overall and disease-specific or disease-free) | NR (2022)  Deaths 48-wks postoperatively:  IG: 7; CG: 14 (2023) | | NR |
| Recurrence (local, regional or distant) | NR (2022)  48-wks postoperatively:  IG:6; CG:5 (2023) | | NR (study authors state that longer follow-up is required) |
| Quality of life (e.g. measured by EQ-5D or SF-36) | NR (2022)  Mean difference (95% CI) (2023)  4 wk 0.002 (–0.008~0.012)  24 wk 0.003 (–0.004~0.010)  48 wk 0.004 (–0.002~0.011) | | NR (study authors state that longer follow-up is required) |
| Time to resume work/daily activities | NR | | NR |
| Patient satisfaction | NR | | NR |
| **Safety-related outcomes** | | | |
| Intraoperative complications (e.g. air-leakage) | NR | | NR |
| Postoperative complications (e.g. infections) | *IG vs CG; n (%); p-value*  **Postoperative complications:**  23 (14.6) vs 30 (18.4); NS  **Clavien Dindo I-II:** 18 (11.5) vs 24 (14.7); NS  Pleural effusion: 8 (5.1) vs 12 (7.4); NS  Pneumoni: 4 (2.5) vs 1 (0.6); NS  Prolonged air leak: 9 (5.7) vs 7 (4.3); NS  Recurrent air leak: 0 vs 1 (0.6); NS  Haemorrhage: 1 (0.6) vs 1 (0.6); NS  Atrial fibrillation: 0 vs 1 (0.6); NS  Ischemic stroke: 0 vs 1 (0.6); NS  Hypoxemia: 0 vs 1 (0.6); NS  **Clavien Dindo III-IV:** 5 (3.2) vs 6 (3.7); NS  Pleural effusion: 2 (1.3) vs 2 (1.2); NS  Pneumonia: 0 vs 1 (0.6), NS  Prolonged air leak: 0 vs 3 (1.8); NS  Recurrent air leak: 1 (0.6) vs 1 (0.6); NS  Haemorrhage: 1 (0.6) vs 1 (0.6); NS  Ischemic stroke: 2 (1.3) vs 0; NS  **Readmission:** 3 (1.9) vs 3 (1.8); NS | | *IG vs CG; n (%); p-value*  **Early postoperative complications^^[[3]](#footnote-3)^^:** 13 (37) vs 9 (24); NS  Air leak: 6 (17) vs 4 (11); NS  Atrial Fibrillation: 4 (11) vs 3 (9); NS  Serious drainage: 1 (3) vs 1 (3); NS  Pneumonia: 4 (11) vs 1 (3); NS  Pneumothorax: 0 (0) vs 1 (3); NS  Atelectasis: 3 (9) vs 1 (3); NS  Urinary tract infection: 1 (3) vs 0 (0); NS  Other Complication: 3 (9) vs 2 (5); NS  **Readmissions:** 4 (16) vs 0 (0); NS  **Later Complication:** 5(23) vs 2 (11); NS |
| Reoperations/additional surgeries | NR | | NR |
| Conversion | *IG vs CG; n (%); p-value*  Conversion to thoracotomy: 7 (4.5) 9 (5.5); NS | | *IG vs CG; n (%); p-value*  Conversion to open surgery: 3 (9) vs 1 (3); NS |
| **Perioperative events & resource use** | | | |
| Blood loss (in ml) | *IG vs CG, median (IQR); p-value*  100 (50–100) vs 100 (50–150); **p= 0.04** | | NR |
| Operation time in min. | *IG vs CG, median (IQR), p-value*  110 (95–140) vs 120 (97.5–150); NS | | *IG vs CG, mean±SD, p-value*  179±54.2 vs 183±40.9; NS |
| Transfusions | *IG vs CG, no. (%), p-value*  Intraoperative blood transfusion:  3 (1.9) vs 2 (1.2); NS | | NR |
| Drain duration (days) | *IG vs CG [mL], median (IQR); p-value*  Chest tube drainage:  830 (550–1,130) vs 685 (367.5–1,160) **p=0.007** | | NR |
| Length of hospital stay (days) | *IG vs CG, median (IQR); p-value*  4 (4–5) vs 5 (4–5); NS | | IG vs CG, median (IQR); p-value  5 (4-8) vs 4 (3-6); NS |
| **Author, year** | **Huang et al. 2019** | **Huang et al. 2021** | **Terra et al. 2022** |
| **Study characteristics** | | | |
| Study design, indication | Noninferiority, phase 3, multi-centre RCT of patients with single cN2 stage NSCLC | | Two-arm randomised clinical trial of patients with lung lesions |
| Country | China | | Brazil |
| Funding/Sponsor | Shanghai Hospital Development Center, National Natural Science Foundation of China | | The Brazilian Ministry of Health |
| Intervention (IG) \| Product | Robot-assisted thoracoscopic surgery \| da Vinci Surgical System | | Robotic-assisted thoracic surgery\| da Vinci Si |
| Comparator (CG) | Thoracotomy | | Video-assisted thoracic surgery |
| Experience of surgeon(s); time period | NR; January 2016 to December 2018 (trial performance) | NR; January 2016 to July 2020 (enrollment) | NR;  April 2015 to June 2017 (trial length) |
| Number of randomised patients | 113; IG: 58; CG:55 | 159; IG: 79; CG: 78 | 80; IG: 40; CG: 40 |
| Inclusion criteria | - 18-75 yrs - Clinically diagnosed cN2 NSCLC according to American Joint Committee on Cancer Tumor-Node-Metastasis classification exhibited as a suspicious pulmonary lesion with enlarged mediastinal LN - Adequate organ function to tolerate pulmonary resection | | - Eligibility or the treatment of lung cancer or lung metastasis by pulmonary lobectomy - presence of a tumour of less than 5 cm in diameter - absence of tumour invasion into the chest wall, diaphragm, mediastinum, or another lung lobe - clinical and anaesthetic evaluation results showing that the patient was able to undergo the proposed procedure |
| Primary/secondary endpoints | - operative time, intraoperative blood loss, chest tube duration, drainage at postoperative day one and total drainage, length of hospital day, death (within 28 days), complications, visual analogue score at postoperative day one to five, overall cost, pathological variables | - primary: disease-free survival, overall survival - secondary: operative duration, blood loss volume, drainage duration, total drainage volume, length of stay, overall cost, pain visual analogue scale score (postoperative days 1-5), postoperative complications | - primary: complication rate within 90 days, postoperative complications - secondary: intraoperative complications, drainage time, length of hospital stay, postoperative pain, postoperative QoL and readmissions within 90 days |
| Follow-up (months) | Only 28 days of follow-up reported  2 yrs after surgery (3-month intervals) (NR)  5 yrs after surgery (6-month intervals) (NR) | 2 yrs after surgery (3-month intervals)  Thereafter 6 months intervals | 90 days after surgery |
| Dropouts (n (%)) | None | After randomisation:  IG: 3 (3.8%); CG: 6 (7.7%) | After randomisation:  IG: 3 (7.5%); CG: 1 (2.5%)  At 90-day follow-up:  IG: 1 (2.5%); CG: 1 (2.5%) |
| **Patient characteristics** | | | |
| Age of patients (yrs., mean) | *IG vs CG; mean (±SD); p-value* | | *IG vs CG; median (95% CI);* p=NS  68.4 (65.2-71.5) vs 65.7 (61.8-69.5) |
|  | 61.9 (±9.0) vs 60.6 (±7.4); NS | 60.9 (±9.4) vs 61.0 (±7.6); NS |  |
| Sex (% female) | *IG vs CG; %; p-value* | | IG: 54%; CG: 56.4%; NS |
|  | 29.3% vs 29.1%; NS | 32.9% vs 29.2%; NS |  |
| BMI (kg/m², mean) | NR | | IG vs CG; median (95% CI); p=NS  27.5 (26.2-28.8) vs 26.5 (24.9-28.1) |
| Clinical classification | *IG vs CG; n (%) ;* *p-value* | | NR |
|  | Pathologic stage:  IA: 10 (17.3) vs 10 (18.2); NS  IB: 8 (13.8) vs 5 (9.0); NS  IIA: 4 (7.0) vs 2 (3.7); NS  IIB: 14 (24.1) vs 10 (18.2); NS  IIIA: 14 (24.1) vs 19 (34.6); NS  IIIB: 6 (10.3) vs 7 (12.8); NS  IV: 2 (3.4) vs 2 (3.5); NS | Pathological TNM stage:  I: 24 (31.6) 21 vs (29.2); NS  II: 24 (31.6) vs 17 (23.6); NS  III: 27 (35.5) vs 33 (45.8); NS  IV: 1 (1.3) vs 1 (1.4); NS |  |
| **Patient-relevant outcomes** | | | |
| Survival (overall and disease-specific or disease-free) | Mortality within 28 days after surgery:  IG: 1 (1.7); CG: 0 (0); NS | *IG vs CG; %; p-value*  Disease-free survival:  1 yr: 90.4 vs 86.0; NS  2 yrs: 76.4 vs 74.2; NS  3 yrs: 57.5 vs 49.9; NS  Overall survival:  1 yr: 97.2 vs 97.0; NS  2 yrs: 94.2 vs 93.2; NS  3 yrs: 84.6 vs 74.9; NS | Mortality within 90 days after surgery:  IG 1 (2.7); CG: 1 (2.5); NS |
| Recurrence (local, regional or distant) | NR | | NR |
| Quality of life (e.g. measured by EQ-5D or SF-36) | NR | | NR |
| Time to resume work/daily activities | NR | | NR |
| Patient satisfaction | NR | | NR |
| **Safety-related outcomes** | | | |
| Intraoperative complications (e.g. air-leakage) | NR | | Intraoperative complications:  IG: 0; CG: 3; p=NS  (2 arterial lacerations and 1 venous injury) |
| Postoperative complications (e.g. infections) | *IG vs CG; n (%); p-value* | | *IG vs CG; n (%); p-value*  **Complications within 90 days:** 7 (18.9) vs 14 (35.9); NS  **≥3 complications within 90 days:** 7 (18.9) vs 10 (25.6); NS  **Readmissions within 90 days:** 1 (2.7) vs 8 (20.5); **p=0.029** |
|  | **Complications within 28 days after surgery:**  Any complications: 16 (27.6) vs 21 (38.2); NS  Pulmonary embolism: 1 (1.7) vs 0 (0); NS  Bronchopleural fistula: 3 (5.2) vs 1 (1.8); NS  Oesophagus fistula: 0 (0) vs 1 (1.8); NS  Acute respiratory distress syndrome: 0 (0) vs 1 (1.8); NS  Pneumonia: 3 (5.2) vs 6 (10.9); NS  Prolonged air leak: 4 (6.9) vs 6 (10.9); NS  Atrial arrhythmia: 2 (3.4) vs 3 (5.5); NS  Chest tube reinsertion: 2 (3.4) vs 3 (5.5); NS  Chylothorax: 3 (5.2) vs 0 (0); NS  Recurrent nerve injury: 1 (1.7) vs 4 (7.3); NS  Others: 1 (1.7) vs 2 (3.6); NS | Prolonged air leak: 6 (7.9) vs 6 (8.3); NS  Bronchopleural fistula: 4 (5.3) vs 1 (1.4); NS  Pneumonia: 3 (3.9) vs 6 (8.3); NS  Atrial fibrillation: 3 (3.9) vs 4 (5.6); NS  Atrial arrhythmia: 3 (3.9) vs 4 (5.6); NS  Chest tube reinsertion: 3 (3.9) vs 4 (5.6); NS  Subcutaneous emphysema: 3 (3.9) vs 2 (2.8); NS  Chylothorax: 3 (3.9) vs 2 (2.8); NS  Hyperpyrexia: 2 (2.6) vs 6 (8.3); NS  Haemorrhage: 2 (2.6) vs 1 (1.4); NS  Recurrent laryngeal nerve injury: 1 (1.3) vs 4 (5.6); NS  Pulmonary embolism: 1 (1.3) vs 0; NS  Pyothorax: 0 vs 1 (1.4); NS  Acute respiratory distress symptom: 0 vs 1 (1.4); NS |  |
| Reoperations/additional surgeries | *IG vs CG; n (%);* p-value  Haemorrhage required reoperation (within 28 days): 1 (1.7) vs 1 (1.8); NS | | *IG vs CG; n (%); p-value*  1 (2.7) vs 2 (5.1); NS |
| Conversion | Conversion to open surgery:  IG: 5 (8.6%); CG: 0 | Conversion to video-assisted thoracic surgery:  IG: 1 (1.3%); CG:0 | Conversion to open surgery:  IG: 0; CG: 2; NS |
| **Perioperative events & resource use** | | | |
| Blood loss (in ml) | *IG vs CG; mean (±SD); p-value*  86.3 (±41.1) vs 165.7 (±46.4); **p<0.001** | *IG vs CG; n (%); p-value*  <100ml: 65 (85.5) vs 16 (22.2); **p<0.001**  ≥100ml: 11 (14.5) vs 56 (77.8); **p<0.001** | NR |
| Operation time in min. | *IG vs CG; mean (±SD); p-value* | | *IG vs CG; median (95% CI); p-value*  241.7 (218.3-265.1) vs 214.4 (200.3-228.5); NS |
|  | 108 (±39) vs 103 (±30); NS | 104.2 (±41.0) vs 102.3 (±29.2); NS |  |
| Transfusions | NR | | IG: 0; CG: 0; p=NR |
| Drain duration (days) | *IG vs CG; mean in mL (range); p-value*  1 day postoperative: 300 (95–840) vs 320 (50–970); NS  Total drainage: 820 (220–2,460) vs 960 (320–4,630); p=0.05 | *IG vs CG; median (IQR); p-value*  Drainage duration (days):  4.0 (3.3–5.0) vs 5.0 (4.0–7.0); **p=0.002**  Total drainage volume: (ml)  855.0 (602.5–1,167.5) vs 920.0 (592.5–1,646.3); NS | *IG vs CG; median (IQR); p-value*  Chest tube:  2 (1-2) vs 2 (1-4); NS |
| Length of hospital stay (days) | *IG vs CG; mean (range); p-value*  10 (7-31) vs 11 (6-44); NS | *IG vs CG; median (IQR); p-value*  10.0 (8.0–13.0) vs 11.0 (9.0–14.8); p=0.054 | *IG vs CG; median (IQR); p-value*  3 (2-4) vs 4 (2-5); NS |

*Table A2: Extraction tables Oesophagus*

| **Oesophagus - Antireflux/Fundoplication** | |
| --- | --- |
| **Author, year** | **Lang et al. 2022** |
| **Study characteristics** | |
| Study design, indication | Randomised controlled trial of patients with gastroesophageal reflux disease |
| Country | Germany |
| Funding/Sponsor | Projekt DEAL |
| Intervention (IG) \| Product | Robotic-assisted laparoscopic fundoplication \| da Vinci Surgical System |
| Comparator (CG) | Conventional laparoscopic fundoplication |
| Experience of surgeon(s); time period | Robotic-assisted / Conventional laparoscopy: >30 surgeries before;-August 2004 to December 2005 (randomisation) |
| Number of randomised patients | 40; IG: 20; CG: 20 |
| Inclusion criteria | - >18 yrs - History of gastroesophageal reflux disease requiring an acid suppressive therapy within proton pump inhibitor for at least 3 months during the preceding year - Disease was initially diagnosed by the presence of endoscopic oesophagitis or by severe clinical symptoms, which resolved with PPI therapy (positive PPI test) and was confirmed by gastrointestinal endoscopy, barium swallow and 24-h pH monitoring |
| Primary/secondary endpoints | - QoL and reflux-specific symptoms |
| Follow-up (months) | 12 yrs |
| Drop-outs (n (%)) | IG: 5 (25%); CG: 5 (25%) |
| **Patient characteristics^^[[4]](#footnote-4)^^** | |
| Age of patients (yrs., mean) | *IG vs CG; mean ± SD (range);* p=NS  49.6 ± 12.0 (23–71) vs 50.5 ± 12.4 (25–75) |
| Sex (% female) | IG: 50%; CG: 60%; p=NS |
| BMI (kg/m², mean) | *IG vs CG; mean ± SD (range);* p=NS  29.2 ± 5.83 (21–40) vs 26.2 ± 3.4 (19–31) |
| Clinical classification | Oesophagitis; p=NS  Los Angeles A: IG:9; CG:11  Los Angeles B: IG: 10; CG: 7  Los Angeles C: IG: 1; CG: 2  Los Angeles D: IG: 0; CG: 0  Gastrointestinal symptom rating scale; p=NS  IG: 4.0 ± 1.7 (2–7); CG: 4.4 ± 1.5 (2–7) |
| **Patient-relevant outcomes** | |
| Survival (overall and disease-specific or disease-free) | NR |
| Recurrence (local, regional or distant) | IG vs CG; n (%); p=NR  **Failure of treatment:**  Oesophagitis ≥LA-B: 1 (8) vs 1 (8)  GSRS reflux score ≥3: 3 (25) vs 2 (17)  Daily PPI for reflux: 4 (31) vs 4 (33)  Dysphagia combined with reflux score ≥2: 1 (8) vs 1 (8) |
| Quality of life (e.g. measured by EQ-5D or SF-36) | *IG vs CG; mean ± SD (range);* p=NS  Quality of life in reflux and dyspepsia:  Emotional distress: 6.4 ± 1.4 (1.2–7.0) vs 6.5 ± 1.6 (1.0–7.0)  Food/drink problems: 6.5 ± 0.9 (3.5–7.0) vs 6.3 ± 1.6 (1.0–7.0)  Physical/social functioning: 6.6 ± 1.0 (2.8–7.0) vs 6.4 ± 1.6 (1.0–7.0)  Sleep disturbance: 6.4 ± 1.3 (2.2–7.0) vs 6.5 ± 1.5 (1.0–7.0)  Vitality: 6.3 ± 1.4 (1.3–7.0) vs 6.3 ± 1.6 (1.0–7.0) |
| Time to resume work/daily activities | NR |
| Patient satisfaction | NR |
| **Safety-related outcomes** | |
| Intraoperative complications (e.g. air-leakage) | NR |
| Postoperative complications (e.g. infections) | NR |
| Reoperations/additional surgeries | *IG vs CG; n (%);* n=NR  Reoperation for reflux 0 (0) vs 0 (0) |
| Conversion | NR |
| **Perioperative events & resource use** | |
| Blood loss (in ml) | NR |
| Operation time in min. | *IG vs CG; mean ± standard deviation* *(range); p-value*  ***Total operative time******^^[[5]](#footnote-5)^^:***  88 ± 18 (60–150) vs 102 ± 19 (75–152) **p=0.033** |
| Transfusions | NR |
| Drain duration (days) | NR |
| Length of hospital stay (days) | *IG vs CG; mean ± standard deviation (range); p-value*  1,710 ± 488 (600–2,400) vs 1,980 ± 481 (1,200–3,000); NS |

| **Oesophagus - Oesophagectomy** | | |
| --- | --- | --- |
| **Author, year** | **De Groot et al. 2020** | **Yang et al. 2022** |
| **Study characteristics** | | |
| Study design, indication | Single-centre randomised controlled trial of patients with intrathoracic oesophagal cancer | Prospective, multicentre, randomised, controlled clinical trial of patients with oesophagal squamous cell carcinoma |
| Country | The Netherlands | China |
| Funding/Sponsor | NR | Shanghai Hospital Development Center |
| Intervention (IG) \| Product | Robot-assisted minimally invasive oesophagectomy \| NR | Robot-assisted minimally invasive oesophagectomy \| da Vinci |
| Comparator (CG) | Open transthoracic oesophagectomy | Conventional minimally invasive oesophagectomy |
| Experience of surgeon(s); time period | ≥50 robotic-assisted and ≥conventional procedures before;  January 2012 and August 2016 (randomisation) | >40 procedures of robotic-assisted or conventional procedures annually;  August 2017 to December 2019 (eligibility assessment and randomisation) |
| Number of randomised patients | 112; IG: 56; CG: 56 | 362; IG: 183; CG: 179 |
| Inclusion criteria | - Age between 18 and 80 - Histologically proven, surgically resectable oesophageal cancer (cT1-4a, N0–3, M0) | - 18-75 yrs - European Clinical Oncology Group performance status of 0, 1, or 2, with primarily resectable oesophageal squamous cell carcinoma of the intrathoracic oesophagus |
| Primary/secondary endpoints | - primary: overall and disease-free survival rates during a follow-up period of 5 years after surgery - secondary: location of disease recurrences | - primary: overall survival - secondary: perioperative outcomes, long-term survival |
| Follow-up (months) | 5 yrs | Every 3 months within the first yr |
| Drop-outs (n (%)) | After 5 yrs follow-up:  IG: 2 (3.6%); CG: 3 (5.4%) | Until 90 days after surgery:  IG: 2 (1.1); CG: 2 (1.1) |
| **Patient characteristics** | | |
| Age of patients (yrs., mean) | *IG vs CG; mean (±SD);* p=NS  64 (±8.9) vs 65 (±8.2) | *IG vs CG; median (range);* p=NS  65 (43–75) vs 63 (42–75) |
| Sex (% female) | IG: 15%; CG: 24%; p=NS | IG: 13.8%; CG: 15.3%; p=NS |
| BMI (kg/m², mean) | *IG vs CG; mean (±SD);* p=NS  26.1 (±4.4) vs 25.5 (±4.7) | *IG vs CG; mean (±SD);* p=NS  23.1 (± 2.8) vs 23.0 (± 3.1) |
| Clinical classification | *IG vs CG; n (%);*p=NS  Clinical Stadium  I-II: 20 (37) vs 25 (45)  III-IV: 34 (63) vs 30 (55)  ASA score  1: 13 (24) vs 11 (20)  2: 38 (70) vs 34 (62)  3: 3 (6) vs 10 (18) | *IG vs CG; n (%);* p=NS  Clinical Stage  I: 28 (15.5) vs 22 (12.4)  II: 94 (51.9) vs 93 (52.5)  III: 57 (31.4) vs 62 (35.0)  Iva: 2 (1.1) vs 0 (0) |
| **Patient-relevant outcomes** | | |
| Survival (overall and disease-specific or disease-free) | *IG vs CG; median in months (range); rate (95% CI);* p=NS  Overall survival: 35 (1–60); 41% (95% CI 27–55) vs 41 (2–60); 40% (95% CI 26–53)  Disease-free survival: 28 (0–56); 42% (95% CI 28–55) vs 37 months (3–56); 43% (95% CI 29–57) | *IG vs CG; n (%);* p=NS  **In-hospital mortality:** 0 (0) vs 0 (0)  30-d mortality: 0 (0) vs 1 (0.6)  90-d mortality: 1 (0.6) vs 1 (0.6) |
| Recurrence (local, regional or distant) | *IG vs CG; n (%);* p=NS  **Overall recurrence disease:** 28 (56) vs 29 (54)  **Anastomoses/gastric conduit:** 3 (6) vs 1 (2)  **LN:** 14 (28) vs 15 (28)  Only inside resection area: 4 (8) vs 3 (6)  Only outside resection area: 3 (6) vs 5 (9)  Both: 7 (14) vs 7 (13)  **Distant:** 26 (52) vs 27 (50)  Liver: 6 (12) vs 12 (22)  Lung: 5 (10) vs 4 (7)  Bone: 5 (10) vs 5 (9)  Pleural: 7 (14) vs 5 (9)  Soft tissue: 4 (8) vs 4 (7)  Peritoneal: 3 (6) vs 3 (6)  Adrenal: 3 (6) vs 5 (9)  Cerebral: 3 (6) vs 2 (4) | NR |
| Quality of life (e.g. measured by EQ-5D or SF-36) | NR | NR |
| Time to resume work/daily activities | NR | NR |
| Patient satisfaction | NR | NR |
| **Safety-related outcomes** | | |
| Intraoperative complications (e.g. air-leakage) | NR | *n (%); conversion reasons*  Conversion to open surgery:  **IG:** 7 (3.9)  Adhesions: 4  Intraoperative bleeding: 2  Unstable circulation: 1  **CG:** 6 (3.4)  Tissue adhesions: 3  Injury of right gastroepiploic artery: 2  Torsional conduit: 1 |
| Postoperative complications (e.g. infections) | NR | *IG vs CG; n (%);* p=NS  **Total complications:** 88 (48.6) vs 74 (41.8)  C-D classification ≥III: 22 (12.2) vs 18 (10.2)  **Pulmonary complications:** 25 (13.8) vs 26 (14.7)  Pneumonia: 18 (9.9) vs 21 (11.9)  Respiratory failure: 8 (4.4) vs 9 (5.1)  Pleural effusion: 10 (5.5) vs 6 (3.4)  Pneumothorax: 3 (1.7) vs 5 (2.8)  **Severe cardiac complications:** 2 (1.1) vs 1 (0.6)  **Anastomotic leakage:** 22 (12.2) vs 20 (11.3)  Type I (conservative): 8 (4.4) vs 5 (2.8); NR  Type II (nonsurgical intervention): 13 (7.2) vs 14 (7.9); NR  Type III (surgical intervention): 1 (0.6) vs 1 (0.6); NR  **Vocal cord paralysis:** 59 (32.6) vs 48 (27.1)  Type I (transient injury requiring no therapy): 55 (30.4) vs 45 (25.4); NR  Type II (requiring elective surgical procedure): 1 (0.6) vs 1 (0.6); NR  Type III (requiring acute surgical intervention): 3 (1.7) vs 2 (1.1); NR  Location (Left/right/bilateral): 49 (27.1)/6 (3.3)/4 (2.2) vs 41 (23.2)/4 (2.3)/3 (1.7)  **Chylothoraxy:** 5 (2.8) vs 2 (1.1)  Type I (enteric dietary modifications): 4 (2.2) vs 1 (0.6); NR  Type II (total parenteral nutrition): 1 (0.6) vs 1 (0.6); NR  Type III (interventional or surgical therapy): 0 (0) vs 0 (0); NR  **Wound infections:** 3 (1.7) vs 1 (0.6)  **Readmission intensive care unit:** 3 (1.7) vs 3 (1.7) |
| Reoperations/additional surgeries | NR | NR |
| Conversion | NR | *IG vs CG; n (%)*; p=NS  7 (3.9) vs 6 (3.4) |
| **Perioperative events & resource use** | | |
| Blood loss (in ml) | NR | *IG vs CG; median (IQR)*; p=NS  200 (100–400) vs 200 (100–500) |
| Operation time in min. | NR | *IG vs CG; mean ± SD*  203.8 ± 59.4 vs 244.9 ± 61.0; **p<0.001** |
| Transfusions | NR | NR |
| Drain duration (days) | NR | Thoracic drainage tube was generally  removed on postoperative day 3 or 4. |
| Length of hospital stay (days) | NR | *IG vs CG; median (range);* p=NS  Postoperative hospital stay:  9 (6–49) vs 9 (6–82) |

*Table A3: Extraction table Stomach*

| **Stomach - Gastrectomy** | | | |
| --- | --- | --- | --- |
| **Author, year** | **Lu et al. 2021** | **Ojima et al. 2021** | **Ribeiro et al. 2022** |
| **Study characteristics** | | | |
| Study design, indication | Open-label, non-inferiority RCT of patients with gastric cancer | Phase 3, prospective superiority RCT of patients with gastric cancer | Prospective, single-institution, open-label, non-inferiority RCT of patients with gastric cancer |
| Country | China | Japan | Brazil |
| Funding/Sponsor | Joint funds for the innovation of science and technology, Fujian province; the second batch of special support funds for Fujian Province innovation and entrepreneurship talents; Construction Project of Fujian Province Minimally Invasive Medical Center; Natural Science Foundation of Fujian Province; Fujian provincial science and technology innovation joint fund project plan; Fujian provincial health technology project | NR | By the Institution (Department of Gastroenterology, Instituto do Cancer do Estado de São Paulo, Hospital das Clinicas, Faculdade de Medicina, Universidade de São Paulo) |
| Intervention (IG) \| Product | Robotic distal gastrectomy \| da Vinci robotic system | Robotic gastrectomy \| da Vinci Si and da Vinci Xi | Robotic gastrectomy \| da Vinci Si |
| Comparator (CG) | Laparoscopic distal gastrectomy | Laparoscopic gastrectomy | Open gastrectomy |
| Experience of surgeon(s); time period | >300 laparoscopic and >50 robotic procedures before;  September 2017 to January 2020 (study conducted) | NR;  April 2018 to October 2020 (enrollment) | Surgeons certified as console surgeons in the da Vinci platform by Intuitive;  February 2015 to December 2020 (study inclusion) |
| Number of randomised patients | 300; IG: 150; CG: 150 | 241; IG: 119; CG: 122 | 65; IG: 33; CG: 32 |
| Inclusion criteria | - 18-75 yrs - Histologically proven gastric cancer, with clinical stage cT1–4aN0/þM0 by preoperative evaluation | - 20-90 yrs - Histologically proven gastric carcinoma - resectable gastric cancer according to the eighth edition of the TNM classification - not applicable for endoscopic submucosal dissection according to the Japanese classification - Eastern Cooperative Oncology Group performance status of 0 or 1 - BMI of less than 35 - no history of gastrointestinal surgery that may affect protocol surgery - no history of chemotherapy or radiotherapy - normal function of major organs | - 18-80 yrs - Histologically confirmed gastric adenocarcinoma - Tumour stage cT1-4a and cN0-1, cM0 (preoperative staged by upper digestive endoscopy, abdominal computed tomography scan + / − endoscopic ultrasound); potentially curative intent gastrectomy; performance status by the Eastern Cooperative Oncology Group of 0 or 1; and (ASA) score up to III. |
| Primary/secondary endpoints | - primary: 3-yr disease-free survival rate - secondary: short-term clinical outcomes including intraoperative outcomes, postoperative recovery course, morbidity, quality of lymphadenectomy, adjuvant chemotherapy completion status, cost difference | - primary: incidence of postoperative intra-abdominal infectious complications - secondary: surgical results (operation time, blood loss, transition rate to open or laparoscopic surgery, and the number of retrieval lymph nodes), postoperative courses (times to resumption of drinking and eating, postoperative hospital stay), and oncologic outcomes (overall survival and disease-free survival), rate of conversion | - primary: short-term surgical outcome; incidence of postoperative intra-abdominal infectious complications (surgical duration, blood loss, number of harvested lymph nodes, R0 resection) - secondary: postoperative complications, hospital length of stay, 90-day readmissions, oncologic outcomes, and surgical mortality (death until 90 days after the procedure or during postoperative hospital stay) |
| Follow-up (months) | 30 days  Only disease-free survival rate: 3 yrs | 12 months (adjuvant chemotherapy) for patients with pathologic stage II or III | 90 days, longer follow-up is planned |
| Drop-outs (n (%)) | After randomisation:  IG: 9 (6%); CG: 8 (5.3%) | IG: 6 (5); CG: 5 (4.1) | After randomisation:  IG: 4 (12.1%); CG: 1 (3.1%) |
| **Patient characteristics** | | | |
| Age of patients (yrs., mean) | *IG vs CG; mean (±SD); p-value*  59.4 (±10.2) vs 59.3 (±11.3); NS | *IG vs CG; median (range); p-value*  71 (34-90) vs 72 (40-90); NR | *IG vs CG; mean (SD); p-value*  59.3 (11.3) vs 58.1 (11.3); NS |
| Sex (% female) | IG: 33.3%; CG: 36.6%; p=NS | IG: 37.6%; CG:35.3%; p=NR | IG: 51.7%; CG: 35.5%; p=NS |
| BMI (kg/m², mean) | *IG vs CG; mean (±SD); p-value*  23.2 (±3.0) vs 22.7 (±3.3); NS | *IG vs CG; median (range); p-value*  21.9  (14.0-32.1) vs 22.4 (14.0-31.9); NR | *IG vs CG; mean (SD); p-value*  23.8 (3.6) vs 23.5 (2.9); NS  *IG vs CG;n (%); p-value*  <25: 20 (69) vs 21 (67.7); NS  25-30: 8 (27.6) vs 9 (29); NS  >30: 1 (3.4) vs 1 (3.2); NS |
| Clinical classification | *IG vs CG; n (%); p-value*  ASA  I: 19 (13.5) vs 20 (14.1); NS  II: 112 (79.4) vs 110 (77.5); NS  III: 10 (7.1) vs 12 (8.5); NS | *IG vs CG; n (%); p-value*  ASA  I: 39 (33.3) vs 44 (37.0); NR  II: 74 (63.2) vs 72 (60.5); NR  III: 4 (3.4) vs 3 (2.5); NR  pT stage  T1a: 25 (21.4) vs 23 (19.3); NR  T1b: 43 (36.8) vs 40 (33.6); NR  T2: 10 (8.5) vs 14 (11.8); NR  T3: 25 (21.4) vs 26 (21.8); NR  T4a: 11 (9.4) vs 15 (12.6); NR  T4b: 3 (2.6) vs 1 (0.8); NR  pN stage  N0: 76 (65.0) vs 70 (58.8)  N1: 19 (16.2) vs 24 (20.2)  N2: 15 (12.8) vs 12 (10.1)  N3: 7 (6.0) vs 13 (10.9) | *IG vs CG; n (%); p-value*  ASA  I: 1 (3.4) vs 1 (3.2); NS  II: 24 (82.8) vs 25 (80.6); NS  III: 4 (13.8) vs 5 (16.1); NS  cT  T1: 8 (27.6) vs 6 (19.4); NS  T2: 6 (20.7) vs 11 (35.5); NS  T3: 14 (48.3) vs 11 (35.5); NS  T4: 1 (3.4) vs 3 (9.7); NS  cN  cN0: 21 (72.4) vs 25 (80.6); NS  cN+: 8 (27.6) vs 6 (19.4); NS  cTNM  I: 14 (48.3) vs 14 (45.2); NS  II: 15 (51.7) vs 16 (51.6); NS  III: 0 (0) vs 1 (3.2); NS |
| **Patient-relevant outcomes** | | | |
| Survival (overall and disease-specific or disease-free) | *IG vs CG; n (%); p-value*  **In-hospital mortality within 30 days postoperative:**  0 (0) vs 0 (0); NA | *IG vs CG; n (%); p-value* (per-protocol analysis)  **Mortality:**  IG: 0; CG: 0; p=NS | **Mortality^^[[6]](#footnote-6)^^:**  IG: 0; CG: 0; p=NR |
| Recurrence (local, regional or distant) | NR | NR | NR |
| Quality of life (e.g. measured by EQ-5D or SF-36) | NR | NR | NR |
| Time to resume work/daily activities | NR | NR | NR |
| Patient satisfaction | NR | NR | NR |
| **Safety-related outcomes** | | | |
| Intraoperative complications (e.g. air-leakage) | NR | NR | IG: 0; CG: NR; p=NR |
| Postoperative complications (e.g. infections) | *IG vs CG; n (%); p-value*  **Overall morbidity:** 13 (9.2) vs 25 (17.6); **p=0.039**  **Surgical morbidity:** 5 (3.5) vs 9 (6.3); NS  Abdominal bleeding: 1 (0.7) vs 3 (2.1); NS  Anastomotic leakage: 0 (0.0) vs 1 (0.7); NS  Ileus: 1 (0.7) vs 1 (0.7); NS  Gastroplegia: 0 (0.0) vs 1 (0.7); NS  Wound infection: 1 (0.7) vs 1 (0.7); NS  Peritoneal infection: 3 (2.1) vs 2 (1.4); NS  **Medical morbidity:** 9 (6.4) vs 20 (14.1); **p=0.033**  Pneumonia: 8 (5.7) vs 16 (11.3); NS  Cardiovascluar system: 1 (0.7) vs 1 (0.7); NS  Livery system: 2 (1.4) vs 1 (0.7); NS  Urinary system: 1 (0.7) vs 2 (1.4); NS  Deep vein thrombosis: 0 (0.0) vs 1 (0.7); NS  **Clavien-Dindo classification:**  I: 0 (0.0) vs 0 (0.0); NS  II: 11 (7.8) vs 22 (15.5); NS  IIIa: 0 (0.0) vs 1 (0.7); NS  IIIb: 1 (0.7) vs 1 (0.7); NS  IV: 1 (0.7) vs 1 (0.7); NS  V: 0 (0.0) vs 0 (0.0); NS  **Unplanned readmission:** 2 (1.4) vs 2 (1.4); NS  Peritoneal infection: 1 (0.7) vs 1 (0.7); NS  Pneumonia: 0 (0.0) vs 1 (0.7); NS  Gastroplegia: 1 (0.7) vs 0 (0.0); NS | *IG vs CG; n (%); p-value* (per-protocol analysis)  **Overall complications, ≥grade IIb:** 10 (8.8) vs 23 (19.7); **p=0.02**  **Overall complications, ≥grade IIIa:** 6 (5.3) vs 19 (16.2); **p=0.01**  **Surgical complications:**  Anastomotic leakage, ≥grade II: 4 (3.5) vs 5 (4.3); NS  Anastomotic leakage, ≥grade IIIa: 3 (2.7) vs 5 (4.3); NS  Pancreatic fistula, ≥grade II: 0 vs 2 (1.7); NS  Pancreatic fistula, ≥grade IIIa: 0 vs 1 (0.9); NS  Intra-abdominal abscess, ≥grade II: 3 (2.7) 3 (2.6); NS  Intra-abdominal abscess, ≥grade IIIa: 2 (1.8) vs 3 (2.6); NS  Intra-abdominal bleeding, ≥grade II: 0 vs 0; NS  Intra-luminal bleeding, ≥grade II: 0 vs 0; NS  Ileus, ≥grade IIIa: 1 (0.9) vs 2 (1.7); NS  Cholecystitis, ≥grade II: 0 vs 3 (2.6); NS  Cholecystitis, ≥grade IIIa: 0 vs 2 (1.7); NS  Hepatic portal venous gas, ≥grade IIIa: 0 vs 1 (0.9); NS  Stenosis, ≥grade IIIa: 0 vs 3 (2.6); NS  Wound infection, ≥grade II: 1 (0.9) vs 1 (0.9); NS  Wound infection, ≥grade IIIa: 1 (0.9) vs 0; NS  **Medical complications:**  Pneumonia, ≥grade II: 1 (0.9) vs 5 (4.3); NS  Pneumonia, ≥grade IIIa: 0 vs 2 (1.7); NS  Pneumothorax, ≥grade IIIa: 0 vs 1 (0.9); NS  Cardiovascular system, ≥grade II: 0 vs 0; NS  Liver system, ≥grade II: 0 vs 0; NS  Urinary system, ≥grade II: 0 vs 1 (0.9); NS  Thrombosis, ≥grade II: 0 vs 0; NS | *IG vs CG; n (%); p-value*  **Postoperative complications (0-30 days postoperative):**  Minor: 4 (13.8) vs 6 (19.4); NS  Major: 4 (13.8) vs 3 (3.2); NS  **Late complications (>30 days postoperative):** 1 (3.4) vs 6 (19.4); NS  **Readmission** (<90 days): 1 (3.4) vs 4 (12.9); NS |
| Reoperations/additional surgeries | *IG vs CG; n (%); p-value*  **Reoperation within 30 days:** 0 (0.0) vs 1 (0.7); NS | *IG vs CG; n (%); p-value* (per-protocol analysis)  **Reoperation, grade IIIb:** 1 (0.9) vs 3 (2.6); NS | **Re-do surgery:**  IG: 0; CG:0; p=NR  **Surgical revision:**  IG: 2; CG: NR; p=NR |
| Conversion | NR | *Conversion type; IG vs CG; n (%); p-value* (per-protocol analysis)  **Overall Conversion:** 4 (3.4) vs 2 (1.7); NS  **Conversion to open:** 2 (NR) vs 2 (NR); NR  **Conversion to laparoscopy:** 2 (NR) vs 0; NR | **Conversion (abdominal incision):**  IG: 2 (6.7%); CG: NR; p=NR |
| **Perioperative events & resource use** | | | |
| Blood loss (in ml) | *IG vs CG; mean (±SD); p-value*  **Intraoperative blood loss:**  41.2 (±45.7) vs 55.7 (±70.5); **p= 0.045** | *IG vs CG; median (range); p-value* (per-protocol analysis)  25 (5-475) vs 25 (5-1,405); NS | *IG vs CG; mean (SD); ,median (IQR); range; p-value*  123.7 (89.3); 111.5 (51.3–153.3); 10–340 vs 276.3 (152.1); 300 (120–400); 40–500; **p<0.001** |
| Operation time in min. | *IG vs CG; mean (±SD); p-value*  187.0 (±32.4) vs 181.6 (±44.4); NS | *IG vs CG; median (range); p-value* (per-protocol analysis)  297 (179-654) vs 245 (131-534); **p=0.001** | *IG vs CG; mean (SD); ,median (IQR); range; p-value*  353.8 (96.4); 358 (282–430.5); 185–509 vs 214.6 (41.6); 200 (185–240); 163–320; **p<0.001** |
| Transfusions | *IG vs CG; n (%); p-value*  **Postoperative transfusion:** 8 (5.7) vs 16 (11.3); NS | *IG vs CG; n (%); p-value* (per-protocol analysis)  **Intraoperative tranfusions:** 1 (0.9) vs 3 (2.6); NS | NR |
| Drain duration (days) | *IG vs CG; mean (±SD); p-value*  **Drainage tube removed time:** 6.5 (±1.8) vs 7.0 (±2.5); NS | A single abdominal drain was inserted into the left subphrenic cavity after reconstruction in both groups. The amylase level in the drainage fluid was checked on postoperative days 1 and 3 (PODs 1 and 3) | **Drainage required:**  IG: 1; CG: 1; NR |
| Length of hospital stay (days) | *IG vs CG; mean (±SD); p-value*  **Postoperative hospital stay:** 7.9 (±3.4) vs 8.2 (±2.5); NS | *IG vs CG; median (range); p-value* (per-protocol analysis)  **Postoperative hospital stay:** 12 (7-43) vs 13 (6-45); NS | *IG vs CG; mean (SD); ,median (IQR); range; p-value*  9.1 (5.5); 7 (6–11); 5.0–30 vs 8.9 (5.6); 7 (5–10); 5.0–27; NS |

*Table A4: Extraction tables Bowel*

| **Bowel - Colectomy** | | |
| --- | --- | --- |
| **Author, year** | **Fleming et al. 2022** | **Park et al. 2019** |
| **Study characteristics** | | |
| Study design, indication | Post hoc analysis of a phase III RCT of patients with cancer or benign colonic pathologies | Prospective randomised study of patients with right-sided colon cancer |
| Country | France | South Korea |
| Funding/Sponsor | No funds, grants, or other support were received during the preparation of this manuscript. | Ministry of Health & Welfare, Republic of Korea |
| Intervention (IG) \| Product | Robotic colectomy \| NR | Robot-assisted right colectomy\| da Vinci Si HD |
| Comparator (CG) | Laparoscopic colectomy | Laparoscopic-assisted right colectomy |
| Experience of surgeon(s); time period | NR; NR | Min. 14 yrs operative experience in practice, >400 laparoscopic procedures (including 40 cases of benign disease), ~30 robotic procedures in colon cancer;  September 2009 to July 2011 (eligibility assessment and randomisation) |
| Number of randomised patients | 127; IG: 43; CG: 84 | 71; IG: 35; CG: 36 |
| Inclusion criteria | - ≥18 - Pts undergoing a right or left colectomy for a malignant or benign pathology - Planned minimally invasive surgery | - ≥18 - Medically cleared for radical right colectomy - Diagnosis confirmed by a colonoscopic biopsy |
| Primary/secondary endpoints | - Adequacy of exposure to the operative field and overall visibility - Surgical morbidity - Anastomotic leak - Resolution of symptoms - Return to bowel function - Pain - Hospital length of stay - Cost-analysis | - primary: length of hospital stay - secondary: morbidity, operation time, 3-yrs disease-free survival |
| Follow-up (months) | NR | 3-month intervals (first 2 yrs)  6-month interval (third to fifth yr) |
| Drop-outs (n (%)) | IG: 0 (0%); CG:0 (0%) | Before Surgery:  IG: 0 (0%); CG: 1 (2.8%)  Lost to follow-up:  IG: 0 (0%); CG: 0 (0%) |
| **Patient characteristics** | | |
| Age of patients (yrs., mean) | *IG vs CG; median (range); p-value*  67 (20–93) vs 65 (22–90); NS | *IG vs CG; mean (SD); p-value*  62.8 (10.5) vs 66.5 (11.4); NS |
| Sex (% female) | IG: 53%; CG: 49%; p=NS | IG: 60.0%; CG: 54.3%; p=NS |
| BMI (kg/m², mean) | *IG vs CG; n (%); p-value*  BMI ≤30: 37 (86) vs 73 (87); NS  BMI >30**:** 6 (14) 11 (13); NS | *IG vs CG; mean (SD); p-value*  24.4 (2.5) vs 23.8 (2.7); NS |
| Clinical classification | *IG vs CG; n (%); p-value*  ASA:  I: 12 (28) vs 27 (32); NS  II: 23 (54) vs 50 (60); NS  III: 8 (19) vs 7 (8); NS | *IG vs CG; n (%); p-value*  ASA:  I: 15 (42.9) vs 21 (60.0); NS  II: 16 (45.7) vs 12 (34.3); NS  III: 4 (11.4) vs 2 (5.7); NS |
| **Patient-relevant outcomes** | | |
| Survival (overall and disease-specific or disease-free) | NR | *IG vs CG; mean (%) (95% CI); p-value*  **Disease-free survival:**  3 yrs after surgery: 88.1 (77.1–99.1) vs 91.1 (81.4–99.9); NS  5 yrs after surgery: 77.4 (60.6–92.1) vs 83.6 (72.1–97.0); NS  **Overall survival:**  3 yrs after surgery: 96.8 (90.6–99.9) vs 94.0 (86.0-99.9); NS  5 yrs after surgery: 91.1 (78.8–99.9) vs 91.0 (81.3–99.9); NS |
| Recurrence (local, regional or distant) | NR | No port site recurrence was noted with a median follow-up of 49 months |
| Quality of life (e.g. measured by EQ-5D or SF-36) | NR | NR |
| Time to resume work/daily activities | NR | NR |
| Patient satisfaction | NR | NR |
| **Safety-related outcomes** | | |
| Intraoperative complications (e.g. air-leakage) | *IG vs CG; n (%); p-value*  3 (7) vs 4 (5); NS | NR |
| Postoperative complications (e.g. infections) | *IG vs CG; n (%); p-value*  **Postoperative surgical complication:** 7 (16) vs 10 (12); NS  **Anastomotic leak:** 2 (5) vs 3 (4); NS  **Medical complication:** 4 (9) vs 8 (10); NS  **Clavien Dindo:**  0: 35 (81) vs 68 (81); NS  I: 3 (7) vs 3 (4); NS  II: 3 (7) vs 7 (8); NS  III: 2 (5) vs 4 (5); NS  IV: 0 (0) vs 2 (2); NS | *IG vs CG;n (%); p-value*  **Perioperative morbidity:** 6 (17.1) vs 7 (20.0); NS  **Wound infection:** 2 (5.6) vs 2 (5.6); NR  **Anastomosis leakage:** 1 (2.8) vs 0 (0); NR  **Intraabdominal abscess:** 0 (0) vs 1 (2.8); NR  **Bleeding:** 1 (2.8) vs 3 (8.5); NR  **Ileus:** 1 (2.8) vs 1 (2.8); NR  **Readmission** (>30 days after discharge): 1 (2.8) vs 2 (5.6); NS |
| Reoperations/additional surgeries | *IG vs CG; n (%); p-value*  2 (5) vs 4 (5); NS | *IG vs CG; n (%); p-value*  **Reoperation (>30 days after discharge):** 1 (2.8) vs 1 (2.8); NS |
| Conversion | *IG vs CG; n (%); p-value*  **Surgeon-reported conversion:**  3 (7) vs 10 (12); NS  Conversion to laparotomy: 2 (4.7) vs 2 (2.5); NR  Conversion to standard pressure: 1 (2.3) vs 8 (9.5); NR  **Extraction site:**  Conversion to laparotomy: 2 (5) vs 2 (2); NR | *IG vs CG; N (%); p-value*  **Conversion to laparotomy:** 0 (0) vs 0 (0); NS |
| **Perioperative events & resource use** | | |
| Blood loss (in ml) | NR | *IG vs CG; mean (SD); p-value*  35.8 (36.3) vs 46.8 (31.3); NS |
| Operation time in min. | *IG vs (CG; median (range); p-value*  172 (107-353) vs 145 (69-380); **p=0.005** | *IG vs CG; mean (SD); p-value*  Skin-to-skin time: 195 (41.0) vs 129.7 (43.2); **p<0.001** |
| Transfusions | NR | NR |
| Drain duration (days) | *IG vs CG; n (%); p-value*  Number of patients requiring drain:  2 (5) vs 6 (7); NS | *IG vs CG; N (%); p-value*  Ileus required a nasogastric drainage before discharge:  1 (2.8) vs 1 (2.8); NR |
| Length of hospital stay (days) | *IG vs CG; median (range); p-value*  3 (2–43) vs 4 (2–15); p=0.05 | *IG vs CG; mean (SD); p-value*  7.9 (4.1) vs 8.3 (4.2); NS |
| **Bowel – Rectal resection** | |  |
| **Author, year** | **Feng et al. 2022a** | **Feng et al. 2022b** |
| **Study characteristics** | | |
| Study design, indication | Single-centre unblinded, parallel‐group, superiority RCT of patients with low rectal cancer | Multicentre, randomised, controlled, unblinded, parallel-group, superiority trial of patients with middle and low rectal cancer |
| Country | China | China |
| Funding/Sponsor | Initiated by the investigators; sponsored by Zhongshan Hospital Fudan University | Shenkang Hospital Development Center, Shanghai Municipal Health Commission (Shanghai), and Zhongshan Hospital Fudan University (Shanghai) |
| Intervention (IG) \| Product | Robotic abdominoperineal resection \| da Vinci S system | Robotic surgery for rectal cancer (e.g. total and mesorectal excision) \| da Vinci Si System |
| Comparator (CG) | Laparoscopic abdominoperineal resection | Conventional laparoscopic surgery |
| Experience of surgeon(s); time period | Surgeons had performed >50 robotic and >50 laparoscopic resections before;  December 2013 – 2016 (randomisation) | Surgeons had performed >100 robotic and >100 laparoscopic surgeries per year; >50 robotic and >50 laparoscopic resections before;  July 2016 – December 2020 (randomisation) |
| Number of randomised patients | 347; IG: 174; CG: 173 | 1,240; IG: 620; CG: 620 |
| Inclusion criteria | - 18-75 yrs - ASA class I−III - histologically confirmed low rectal adenocarcinoma (inferior tumour edge ≤5 cm from the anal verge) - assessed as clinical T1‐T3 (mesorectal fascia not involved), N0‐1 or ycT1‐T3, Nx after preoperative neoadjuvant chemoradiotherapy by pelvic MRI - suitable for both robotic and laparoscopic surgery - no evidence of distant metastases - no other malignancies in the medical history except adequately treated basocellular carcinoma of the skin or in situ carcinoma of the cervix uteri | - 18-80 yrs - ASA class I-III - histologically proven rectal adenocarcinoma - assessed as cT1–T3 (the mesorectal fascia not involved) N0–N1 or ycT1–T3 Nx after preoperative radiotherapy or chemoradiotherapy - single middle or low rectal cancer (inferior tumour edge ≤10 cm from the anal verge, as measured by rigid rectoscopy) - the location of the tumour was categorised as middle (>5 to 10 cm from the anal verge) or low (≤5 cm from the anal verge) - no evidence of distant metastasis - no other malignancies in the medical history - suitable for both robotic and laparoscopic surgery |
| Primary/secondary endpoints | - primary: 30‐day postoperative complication rate (Clavien−Dindo grade II or higher) of the intent‐to‐treat population - secondary: compliance, surgical quality, pathological outcomes, postoperative short‐term recovery, urinary and sexual function, and long‐term oncological outcomes | - primary: 3-yrs locoregional recurrence (any cancer recurrence in the pelvic or perineal area at 3 yrs after surgery - secondary: CRM positivity, 30-day postoperative complications, intraoperative outcomes, pathological outcomes, postoperative recovery |
| Follow-up (months) | 1 month (primary outcome); 3 yrs (36 months) after surgery | 1 month; ongoing |
| Drop-outs (n (%)) | By 30 days after surgery for primary outcome: IG: 0; CG: 0  By 3 yrs after surgery: 17 (4.9%) pts were lost to follow‐up, 40 (11.5%) died | Before surgery: IG: 34; CG 35  Change in groups: IG: 6; CG: 7 |
| **Patient characteristics** | | |
| Age of patients (yrs., mean (SD)) | IG: 58.2 (9.6); CG: 59.5 (10.9) | IG: 59.1 (11.0); CG: 60.7 (9.8) |
| Sex (% female) | IG: 37.9; CG: 34.7 | IG: 39.2; CG: 39.5 |
| BMI (kg/m², n (%)) | - Underweight <18.5: 4 (2.3) vs 10 (5.8) - Normal 18.5−23.9: 107 (61.5) vs 106 (61.3) - Overweight 24−27.9: 56 (32.2) vs 52 (30.1) - Obese ≥28: 7 (4.0) vs 5 (2.9) | - Underweight <18.5: 31 (5.3) vs 32 (5.5) - Normal 18.5–23.9: 296 (50.5) vs 299 (51·1) - Overweight 24–27.9: 213 (36.3) vs 210 (35.9) - Obese ≥28: 46 (7.8) vs 44 (7.5) |
| Clinical classification | TNM (IG vs CG) p=NS  I: 33.3% vs 38.2%  II: 32.2% vs 32.9%  III: 34.5% vs 28.9%  ASA (IG vs CG) p=NS  I: 71.3% vs 67.1%  II: 26.4% vs 28.9%  III: 2.3% vs 4.0% | TNM Stage (IG vs CG) p=NR  I: 35.0% vs 34.7%  II: 32.8% vs 34.2%  III: 32.3% vs 31.1%  ASA (IG vs CG) p=NR  I: 55.3% vs 54.4%  II: 39.2% vs 41.0%  III: 5.5% vs 4.6%  T Stage (IG vs CG) p=NR  I-II: 42.5% vs 42.6%  III: 57.5% vs 57.4%  N Stage (IG vs CG) p=NR  0: 67.7% vs 68.9%  I: 24.9% vs 24.8%  II: 7.3% vs 6.3% |
| **Patient-relevant outcomes** | | |
| Survival (overall and disease-specific or disease-free) | **Disease‐free survival** (3‐yrs rate of stage I−III pts):  85.3% vs 84.6% (log‐rank NS; HR=0.918; 95% CI = 0.555−1.517); NS  **Overall survival** (3‐yrs rate of all pts):  91.1% vs 90.4% (log‐rank NS; HR=0.912; 95% CI = 0.490−1.697); NS | NR |
| Recurrence (local, regional or distant) | *IG vs CG (n(%); difference (95% CI); p-value*  **Recurrence at 3 yrs after surgery** *(***Locoregional recurrence:** 5 (2.9) vs 9 (5.2); -2.3 (-7.0 to 2.1); NS   - **Distant metastases:** 21 (12.1) vs 23 (13.3); -1.2 (-8.3 to 6.0); NS | NR |
| Quality of life (e.g. measured by EQ-5D or SF-36) | NR | NR |
| Time to resume work/daily activities | NR | NR |
| Patient satisfaction | NR | NR |
| **Safety-related outcomes** | | |
| Intraoperative complications (e.g. air-leakage) | *IG vs CG; n (%); p-value*  **Pts with any intraoperative complications:** 10 (5.7) vs 16 (9.2); NS  Damage to organ or structure: 4 (2.3) vs 7 (4.0); NR  Equipment failure: 2 (1.1) vs 0 (0); NR  Iatrogenic tumour perforation: 1 (0.6) vs 3 (1.7); NR  Significant haemorrhage^^[[7]](#footnote-7)^^: 3 (1.7) vs 7 (4.0); NR | *IG vs CG; n (%); difference (95% CI); p-value*  **Intraoperative complications**: 32 (5.5%) vs 51 (8.7%);–3·3 (–6·3 to –0·3); **p=0.030**  **Significant bleeding:** 16 (2.7%) vs 26 (4.4%); –1·7 (–4·0 to 0·4); NS  **Damage to organs or structures:** 8 (1.4%) vs 14 (2.4%); –1·0 (–2·8 to 0·6); NS  **Damage to seminal vesicle gland:** 3 (0.5%) 8 (1.4%); –0·9 (–2·2 to 0·3); NR  **Damage to prostate:** 2 (0.3%) vs 3 (0.5%); –0·2 (–1·2 to 0·8); NR  **Damage to ureter:** 1 (0.2%) vs 2 (0.3%); –0·2 (–1·1 to 0·7); NR  **Damage to the vagina:** 2 (0.3%) vs 1 (0.2%); 0·2 (–0·7 to 1·1); NR  **Anastomotic complications:** 4/486 (0.8%) vs 9/449 (2.0%); –1·2 (–3·0 to 0·4); NS  **Iatrogenic perforation:** 4 (0.7%) vs 5 (0.9%); –0·2 (–1·4 to 1·0); NS  **Equipment failure:** 2 (0.3%) vs 0; 0·3 (–0·3 to 1·2); NS |
| Postoperative complications (e.g. infections) | *IG vs CG: n (%); unadjusted difference (95% CI); p-value (within 30 days after surgery)*  **Total 30-day postoperative complication rate (Clavien−Dindo grade II or higher):**  23 (13.2) vs 41 (23.7); -10.5 (-18.6 to -2.3); **p=0.013**  **Postoperative mortality:** 0 vs 0; NE; NE  **Clavien−Dindo classification grade II:** 16 (9.2) vs 28 (16.2); −7.0 (−14.1 to 0.1); NS  **Clavien−Dindo classification grade III:** 7 (4.0) vs 12 (6.9); −2.9 (−8.1 to 2.1); NS  **Wound infection:** 4 (2.3) vs 4 (2.3); 0 (−3.8 to 3.8); NS  **Ileus:** 1 (0.6) vs 3 (1.7); −1.2 (−4.5 to 1.6); NS  **Abdominal/pelvic infection/abscess:** 0 (0) vs 2 (1.2); −1.2 (−4.1 to 1.0); NS  **Pulmonary infection/pleural effusion:** 1 (0.6) vs 1 (0.6); 0 (−2.7 to 2.7); NS  **Stomal complications:** 1 (0.6) vs 1 (0.6); 0 (−2.7 to 2.7); NS  **Anastomotic leakage:** 0/6 vs 1/3; −33.3 (−80.7 to 18.5); NS  **Clavien−Dindo classification grade IV:** 0 (0) vs 1 (0.6); −0.6 (−3.2 to 1.6); NS  **Readmission within 30 days:** 4 (2.3) vs 12 (6.9); −4.6 (−9.6 to −0.1); **p=0.044**  In the subgroup analysis, more advantages were observed in the IG for male patients, age ≥60 yrs, BMI ≥24 kg/m^2^, tumour size ≥4 cm, and pathological N stage positivity (no statistical significance in the interaction analysis) | *IG vs CG: n (%); difference (95% CI); p-value (within 30 days after surgery)*  **Mortality within 30 days postoperatively**: 1 (0.2) vs 1 (0.2); 0.0 (-0.8 to 0.8); NS  **Complications of Clavien–Dindo grade II or higher grade within 30 days after operation:**  95 (16.2) vs 135 (23.1); -6.9 (-11.4 to -2.3); **p=0.003**  **Anastomotic leakage:** 25/486 (5.1) vs 37/449 (8.2); -3.1 (–6.5 to 0.1); NS  **Abdominal or anastomotic bleeding:** 8 (1.4) vs 12 (2.1); –0.7 (–2.3 to 09); NS  **Wound-related:** 18 (3.1) vs 22 (3.8); –0.7 (–2.9 to 1.5); NS  **Ileus:** 5 (0.9) vs 11 (1.9); –1.0 (–2.6 to 0.3); NS  **Urinary retention or infection:** 10 (1.7) vs 17 (2.9); –1.2 (–3.1 to 0.6); NS  **Stoma-related:** 3/229 (1.3) vs 4/253 (1.6 ;–0.3 (–2.9 to 2.4); NS  **Deep vein thrombosis:** 6 (1.0) vs 9 (1.5); –0.5 (–2.0 to 0.9); NS  **Central venous catheter infection**: 7 (1.2) vs 6 (1.0); 0.2 (–1.2 to 1.5); NS  **Pulmonary infection:** 4 (0.7) vs 7 (1.2); –0.5 (–1.9 to 0.7); NS  **Arrhythmia and hypertension:** 12 (2.0) vs 9 (1.5); 0.5 (–1.1 to 2.2); NS  **Others**: 7 (1.2) vs 9 (1.5); –0·3 (–1.9 to 1.1); NS  **Readmissions within 30 days after operation:** 17 (2.9) vs 20 (3.4); –0.5 (–2.6 to 1.6); NS |
| Reoperations/additional surgeries | *IG vs CG: n (%); unadjusted difference (95% CI); p-value*  **Reoperation within 30 days:** 5 (2.9) vs 10 (5.8); −2.9 (−7.7 to 1.6); NS | *IG vs CG: n (%); difference (95% CI); p-value (within 30 days after surgery)*  **Reoperation within 30 days:** 14 (2.4) vs 24 (4.1); –1.7 (–3.9 to 0.3); NS |
| Conversion | *IG vs CG; n (%); p-value*  **Open conversion:** 0 (0) vs 5 (2.9); **p=0.030** | *IG vs CG; n (%); difference (95% CI); p-value*  Conversion to open surgery: 10 (1.7) vs 23 (3.9); –2.2 (–4.3 to –0.4); **p=0.021** |
| **Perioperative events & resource use** | | |
| Blood loss (in ml) | *IG vs CG; median (IQR); p-value*  **Intraoperative haemorrhage:** 100 (90−110) vs 130 (100−150); **p<0.001** | *IG vs CG; median (IQR); difference (95% CI); p-value*  Estimated blood loss: 40.0 (30.0 - 100.0) vs 50.0 (40.0 -100.0); –10·0 (–20.0 to –10.0); **p<0.0001** |
| Operation time in min. | *IG vs CG; median (IQR); p-value*  205 (195−220) vs 195 (160−238); **p=0.004** | *IG vs CG; median (IQR); difference (95% CI); p-value*  173.0 (140.0 - 225.0) vs 170.0 (140.0 – 209.0); 2.0 (–4.0 to 10.0); NS |
| Transfusions | *IG vs CG; n (%); p-value*  **Patients with perioperative transfusion:** 0 (0) vs 1 (0.6); NS | *IG vs CG; n(%);difference (95% CI); p-value*  **Blood transfusions:** 2 (0.3) vs 7 (1.2); -0.9 (-2.2 to 0.2); NS |
| Drain duration (days) | *n (%); p-value*  **Drainage:**  **No drainage tube placed in the abdominal cavity:** 164 (94.3) vs 158 (91.3); NS  **One drainage tube placed in the pelvic cavity through the perineum:** 174 (100) vs 173 (100); NE  **Urinary drainage:**  **Using urinary catheterisation:** 174 (100) vs 173 (100); NE  **Since postoperative day 2, clipping the urinary catheter to exercise bladder function. If patients felt bladder filling, remove the catheter:** 165 (94.8) vs 166 (96.0); NS | NR (pts with grade II anastomotic leakage recovered after fasting, anti-infection measures, nutritional support, and drainage (placed during primary tumour surgery)) |
| Length of hospital stay (days) | *IG vs CG; median (IQR); p-value*  **Hospital stay after surgery:** 5.0 (5.0−6.0) vs 7.0 (6.0−9.0); **p<0.001** | *IG vs CG; median (IQR); difference (95% CI); p-value*  **Postoperative hospital stay**: 7.0 (7.0-11.0) vs 8.0 (7.0-12.0); –1.0 (–1.0 to 0.0); **p=0.0001** |

| **Bowel – Ventral Mesh Rectopexy** | | |
| --- | --- | --- |
| **Author, year** | **Laitakari et al. 2020 & Mäkelä-Kaikkonen et al. 2019** | **Mäkelä-Kaikonen et al. 2016 – Original study from the EUnetHTA report** |
| Study design | Follow-up single-centre randomised controlled trials of patients with external or internal rectal prolapse | Single-centre RCT of patients with rectal prolapse and intussusception |
| Country | Finland | Finland |
| Funding/Sponsor | University of Oulu including Oulu University Hospital | State funding of the Medical Research Center Oulu University and the Finnish Menopause Society |
| Intervention (IG) \| Product | Robot-assisted ventral mesh rectopexy \| da Vinci Si | Robot-assisted ventral mesh rectopexy \| da Vinci Surgical System |
| Comparator (CG) | Laparoscopic ventral mesh rectopexy | Laparoscopic ventral mesh rectopexy |
| Experience of surgeon(s), time period | NR; February 2012 and May 2012 (recruitment) (2019, 2020) | 3 experienced surgeons performed IG; 4 (these + 1 additional surgeon) performed CG. NR: No of prior operations. Operations performed from February to May 2012 |
| Number of patients | 30; IG. 16; CG:14 | IG: 16 (total relapse 4, intrassusception 12)  CG: 14 (total relapse 2, intussusception 11, 1 excluded) |
| Inclusion/exclusion criteria | - 18-85 yrs - Female - External rectal prolapse or recto-anal internal rectal prolapse, with or without the descent of the middle pelvic compartment, combined with symptoms of faecal incontinence and/ or obstructive defaecation   (Details reported in the previous publication) | Inclusion:   - females; - age 18-85; - ASA 1-3; - symptomatic, uncomplicated, isolated, rectal prolapse; symptomatic intussusception and enterocele   Exclusion:   - male; - ASA 4-5; - previous surgery; pregnancy now or future; suspicion of frozen pelvis |
| Primary/secondary endpoints | - primary: maintenance of the repaired pelvic anatomy 5 yrs after surgery (2020) - secondary: persistence of the effect of ventral mesh rectopexy on pelvic anatomy and functional results (2020) - primary: health care costs and HRQoL (2019)   secondary: anatomical outcome and functional outcome (2019) | Perioperative parameters, complications and restoration of anatomy, postoperative pain via VAS |
| Follow-up (months) | 5 yrs (2020); 24 months (2019) | Pain assessment 2 weeks after surgery  Quality of life (Pelvic Floor Distress Inventory, Pelvic Floor Impact Questionnaire, Prolapse/Incontinence Sexual Questionnaire) also condition-specific symptom and quality of life questionnaires (unspecified) at 3 months |
| Drop-outs (n (%) | Until follow-up at 5 yrs postoperative  IG: 2 (12.5%); CG: 2 (14.3%) | QoL data on a total of between 19 and 26 patients; drop-out 35% to 52% |
| **Patient characteristics** | | |
| Age of patients (yrs.) | Overall; mean (SD); p-value  62.5 (11.2); p=NR (2019)  NR (2020); | IG: Ø 60.8 ±11.5  CG: Ø 66.0 ±10.1, p=NR |
| Sex (% female) | IG: 100%; CG: 100% | IG and CG: 100%, p=NR |
| BMI (kg/m²) | NR | IG: Ø 25.6 ±4.5  CG: Ø 24.3 ±3.0, p=NR |
| Clinical classification | NR | ASA (% IG vs. % CG), p=NR  1: 19% vs. 21%  2: 63% vs. 36%  3: 19% vs. 36% |
| **Patient-relevant outcomes** | | |
| Survival (overall and disease-specific or disease-free) | NR | NR |
| Recurrence (local, regional or distant) | At 24-month follow-up (2019):  IG: 0 vs CG: 1 (8%); p=NR | NR |
| Quality of life or symptoms (e.g. measured by EQ-5D or SF-36) | *IG vs CG; n; mean (SD); difference between means (95% CI); p-value*  QoL measurements 5 yrs postoperative (2020):  CRAIQ-7: 14; 24.3 (32.0) vs 10; 43.8 (27.1); -20.4 (-43.2 to 2.5); NS  POPIQ-7: 13; 9.5 (26.4) vs 10; 26.0 (27.9); -16.1 (-39.7 to 7.5); NS  UIQ-7: 14; 25.7 (32.7) vs 10; 33.0 (31.4); -9.4 (-32.3 to 13.6); NS  PFIQ-7: 14; 58.8 (82.1) vs 10; 102.7 (69.9); -47.8 (-103.7 to 8.0); NS | VAS @ 2 weeks:  IG: Ø 2.9 ±1.8  CG: Ø 2.6 ±1.4, p=NS  QoL at 3 months, mean difference (95% CI):  PFDI-20: -61.9 CI_95%_ [40.9 ;82.8%], p<0.01  PFIQ-7: -57.0 CI_95%_ [29.3;84.5%], p<0.01  PISQ-12: 3.4 CI_95%_ [-6.2;-7.6%], p<0.05  No significant differences were found in symptom and condition-specific QoL scores in the between-group comparison as reported for the PFDI and 2 subscales (CRADI and POPDI). No between-group results were reported for PFIQ or PISQ. |
| Time to resume work/daily activities | NR | NR |
| Patient satisfaction | Satisfaction rate: (2019)  IG: 87% vs 69%; NS | NR |
| **Safety-related outcomes** | | |
| Intraoperative complications (e.g. air-leakage) | NR | Perioperative bleeding:  IG 2/16; CG 0/14, p=NS |
| Postoperative complications (e.g. infections) | NR | Vascular complication: IG 1; CG 0, p=NS  Minor complications, p=NS  Haemotoma: IG 1/16; CG 0  Perineal pain: IG 1/16; CG 0  Fever: IG 0; CG 1/14 |
| Re-operations/ additional surgeries | At 24-month follow-up (2019): | NR |
| Conversion | IG: 0 vs CG: 1 (8%); p=NR | IG 0  CG 0 |
| **Perioperative events & resource use** | | |
| Blood loss (in ml) | NR | NR |
| Operation time in min. | IG: 125; CG: 130; p=NS (2019) | IG: Ø 125 ±27  CG: Ø 130 ±25, p=NS |
| Transfusions | NR | NR |
| Drain duration (days) | NR | NR |
| Length of hospital stay (days) | IG: 2.2; CG: 2.5; p=NS (2019) | IG: Ø 2.2 ±1.5  CG: Ø 2.5 ±0.9, p=NS |

*Table A5: Extraction table Gallbladder/Liver/Spleen*

| **Gallbladder/Liver/Spleen – Hernia repair** | | | |
| --- | --- | --- | --- |
| **Author, year** | **Costa et al. 2023** | **Prabhu et al. 2020 & Miller et al. 2023** | **Olavarria et al. 2020** |
| **Study characteristics** | | | |
| Study design, indication | Single-blinded parallel-arm randomised controlled trial of patients with abdominal or pelvic incisional hernia | Multicentre, single-blinded, prospective randomised clinical pilot study (2020) and Follow-up (2023) of patients with inguinal hernia | Multicentre, multi-blinded, randomised controlled trial of patients with ventral hernia defect |
| Country | Brazil | USA | USA |
| Funding/Sponsor | No specific grant from funding agencies in the public, commercial, or not-for-profit sectors. | Intuitive Surgical (IUSI1602MR) | Intuitive Surgical |
| Intervention (IG) \| Product | Robotic-assisted incisional hernia repair \| da Vinci Si | Robotic transabdominal preperitoneal repair \| NR | Robotic ventral hernia repair \| NR |
| Comparator (CG) | Laparoscopic incisional hernia repair | Standard laparoscopic transabdominal preperitoneal repair | Laparoscopic repair |
| Experience of surgeon(s); time period | >50 minimal invasive hernia repairs before; May 2015 to September 2015 (recruitment) | >25 robotic and laparoscopic procedures before; April 2016 to April 2019 (enrollment) | Only surgeons experienced in minimally invasive hernia were allowed to participate in the study;  April 2018 to February 2019 |
| Number of randomised patients | 40; IG: 20; CG:20 | 102; IG: 48; CG: 54 | 124; IG: 65; CG: 59 |
| Inclusion criteria | - adult patients who met the criteria for any abdominal or pelvic incisional hernia | - ≥21 yrs - no prior open abdominal surgery, presenting for primary or recurrent unilateral inguinal hernia repair - no previous preperitoneal mesh placement - BMI ≤40 | - >18 yrs - Ventral hernia defect less than 12cm wide on physical examination, who would likely tolerate pneumoperitoneum - No history of open abdomen or extensive lysis of adhesions for bowel obstruction - No active infection (mesh infection) |
| Primary/secondary endpoints | - primary: length of time in the operating room, operative complications, postoperative length of hospital stay, hernia recurrence at 24-month follow-up - secondary: QoL and abdominal wall strength evaluation | - primary: was not selected because this study was designed as a pilot study (2020) - secondary: cost, surgeon ergonomics, multidimensional workload (2020) | - primary: number of days in hospital at 90 days after surgery (including postoperative and readmission length of stay) - secondary: operating room duration (incision to skin closure time), surgical site infections, surgical site occurrences, hernia recurrence, reoperation, Clavien-Dindo complication grades, emergency department visits, change in abdominal wall QoL, change in visual analogue scale pain scores, and costs from the healthcare system perspective |
| Follow-up (months) | In general: 7 days, 3 months, yearly and 2 yrs after surgery  Hernia recurrence: 24 months after surgery  QoL: 1 month and 24 months after surgery  Abdominal wall strength: 24 months after surgery | 7 days (±3 days) (2020)  1 month (±1 week) (2020)  12 months (± 1 month) after surgery (2023)  24 months (± 1 month) after surgery (2023) | 1 month after surgery  90 days after surgery |
| Drop-outs (n (%)) | Before Surgery:  IG: 2 (10%); CG: 1 (5%) | 30 days after surgery:  IG: 3 (6.3); CG: 1 (1.9); (2020)  2 yrs after surgery:  IG: 14 (29.2%); CG: 11 (20.4%); (2023) | At 90 days after surgery:  IG: 0; CG: 1 (1.7%) |
| **Patient characteristics** | | | |
| Age of patients (yrs., mean) | *mean (SD);* p-value  IG: 65.2 (10.8); CG 59.7 (12.7); NS | *mean (SD);* p-value  IG: 56.1 (14.1); CG: 57.2 (13.3); NS | *IG vs CG; mean (SD); p-value*  50.1 (13.3) vs 48.0 (12.9); NS |
| Sex (% female) | IG: 61.1; CG: 68.4 | IG: 4 (8.4); CG: 6 (11.1%) | IG: 74%; CG: 63%; NS |
| BMI (kg/m², mean) | *mean (SD)*; p-value  IG: 30.5 (4.4); CG: 32.6 (6.6); NS | *mean (SD); p-value*  IG: 24.9 (3.24); CG: 26.9 (4.42); **p=0.014** | *IG vs CG; mean (SD);* *p-value*  32.4 (4.6) vs 31.8 (5.4); NS |
| Disease | NR | NR | *IG vs CG; n (%);* p-value  ASA:  1-2: 42 (65) vs 42 (71); NS  3-4: 23 (35) 17 (29); NS |
| Clinical classification | NR | NR | *IG vs CG; mean (SD); p-value*  50.1 (13.3) vs 48.0 (12.9); NS |
| **Patient-relevant outcomes** | | | |
| Survival (overall and disease-specific or disease-free) | *IG vs CG; n (%); p-value*  Mortality (short-term, within 7 days): 0 vs 1 (5); NS | NR | NR |
| Recurrence (local, regional or distant) | IG: 2 (11.1); CG: 3 (15.75) (in 24-month-follow-up) | *IG vs CG; n;* p=NS  NR (2020)  Inguinal hernia recurrence:  2 yrs after surgery: 1 vs 1 (2023) | IG: 0 (0%); CG: 0 (0%); p=NS |
| Quality of life (e.g. measured by EQ-5D or SF-36) | *IG vs CG; mean (SD); p-value*  Evaluated with the EORTC QLQ-C30  **30 days after surgery:**  Global health: 77.36 (24.06) vs 71.00 (26.15); NS  Functional: 78.93 (23.61) vs 73.36 (21.51); NS  Symptoms: 23.13 (18.55) vs 29.07 (19.26); NS  **2-yrs after surgery:**  Global health: 72.07 (22.67) vs 67.69 (26.32); NS  Functional: 77.27 (19.85) vs 67.19 (21.40); NS  Symptoms: 22.13 (14.72) vs 30 (19.15); NS | *IG vs CG; mean (SD); p-value*  Measured with the SF-36  **1 wk after surgery:**  Physical component summary: -6.95 (8.64) vs -6.52 (8.50); NS  Mental component summary: 0.00 (7.38) vs 0.80 (7.91); NS  General Health: -1.72 (9.57) vs -1.98 (13.4); NS  **30 days after surgery:**  Physical component summary: -1.98 (8.90) vs -0.59 (8.91); NS  Mental component summary: 0.71 (5.84) vs 0.65 (8.29); NS  General Health: 1.55 (8.43) vs -2.31 (12.4); NS  *IG vs CG; mean (SD); p-value*  **1-yr after surgery:**  Physical component summary: 54.9 (7.3) vs 53.7 (8.2); NS  Mental component summary: 55.9 (4.6) vs 54.8 (6.0); NS  General Health: 82.6 (13.1) vs 76.8 (17.7); NS  **2-yrs after surgery:**  Physical component summary: 53.1 (8.1) vs 54.2 (6.1); NS  Mental component summary: 53.9 (6.8) vs 53.4 (5.6); NS  General Health: 77.8 (13.7) vs 77.8 (15.5); NS | *IG vs CG; median (IQR); difference in median (95% CI);* p-value  Abdominal wall QoL measured by the modified Activity Assessment Scale:  52 (37-68) vs 65 (36-86); 8.25 (–1.75 to 20.00); NS |
| Time to resume work/daily activities | NR | NR | NR |
| Patient satisfaction | NR | NR | NR |
| **Safety-related outcomes** | | | |
| Intraoperative complications (e.g. air-leakage) | NR | NR | NR |
| Postoperative complications (e.g. infections) | *IG vs CG; n (%); p-value*  Complications (short-term, within 7 days): 3 (16.7) vs 2 (10.5); NS | *IG vs CG; n (%); p-value*  30-days after surgery:  **Adverse Events:** 8 (16.7) vs 5 (9.3); NS  Superficial surgical site infections: 0 (0.00) vs 1 (1.85); NS  Purulent drainage from the wound: 0 (0.00) vs 1 (1.85); NS  Seroma: 6 (12.5) vs 3 (5.6); NS  Hematoma: 1 (2.08) vs 0 (0.00); NS  Required Intervention: 0 (0.00) vs 1 (1.85); NS  Oral Antibiotics: 0 (0.00) vs 1 (1.85); NS  Urinary retention: 1 (2.08) vs 1 (1.85); NS | *IG vs CG; n (%); relative rate (95% CI); p-value*  **Readmission:** 1 (2) vs 3 (5); 0.27 (0.03 to 2.43); p=NS  **Emergency room visits:** 7 (11) vs 5 (9); 1.28 (0.43 to 3.75); p=NS  **Wound complication:** 13 (20) vs 11 (19); 1.02 (0.51 to 2.08); p=NS  Surgical site infection: 0 (0) vs 1 (2); NR; p=NS  Seroma: 13 (20) vs 8 (14); NR; NS  Hematoma: 0 (0) vs 2 (3); NR; NS  **Clavien-Dindo complication:** 14 (22) vs 11 (19); 1.10 (0.54 to 2.24); NS  1-2: 14 (22) vs 10 (17); NR; NR  3-5: 0 (0) vs 1 (2); NR; NR |
| Reoperations/additional surgeries | None of these patients manifested a desire for reoperation at a 24-month follow-up | NR | *IG vs CG; n (%)*  Reoperation:  IG: 0 (0%); CG: 1 (2%); NS |
| Conversion | No conversion was registered. | One patient in the robotic group was converted to a laparoscopic procedure due to bleeding and was analysed based on intent to treat principles in the robotic group (2020). | *IG vs CG; n (%); relative rate (95% CI);* p-value  Conversion to open repair:  1 (2) vs 1 (2); 0.76 (0.05 to 11.47); NS |
| **Perioperative events & resource use** | | | |
| Blood loss (in ml) | NR | NR | NR |
| Operation time in min. | *IG vs CG; mean (SD); p-value*  355.6 (89) vs 293.5 (89); **p=0.04** | *IG vs CG; median (25^th^; 75^th^); p-value* (2020)  Time from skin incision to closure: 75.5 (59.0; 93.8) vs 40.5 (29.2; 63.8); **p<0.001**  Time for dissection of the hernia: 18.0 (12.0; 27.0) vs 13.0 (7.00; 23.0); **p=0.012**  Time for mesh fixation: 6.88 (5.00; 9.00) vs 1.00 (1.00; 3.00); **p<0.001**  Time for peritoneal closure: 7.00 (5.00; 9.00) vs 2.00 (1.00; 3.00); **p<0.001** | *IG vs CG; mean (SD); relative rate (95% CI); p value*  141 (56) vs 77 (37); 62.89 (45.75 to 80.01); **p<0.001** |
| Transfusions | NR | NR | NR |
| Drain duration (days) | NR | NR | NR |
| Length of hospital stay (days) | *IG vs CG; mean (SD); p-value*  3.67 (1.78) vs 3.95 (2.66); NS | IG vs CG; hours; median (IQR); p=NS  5.75 (5.00; 7.00) vs 5.11 (4.00; 7.00) (2020) | *IG vs CG; n (%); p-value*  Days in hospital at 90 days:  0 days 50: (77) vs 49 (84); NS  1 day 9: (14) vs 4 (7); NS  2 days: 4 (6) vs 1 (2); NS  >3 days: 2 (3) vs 4 (7); NS |

| **Author, year** | **Dhanani et al. 2021** | **Petro et al. 2021 & 2022** |
| --- | --- | --- |
| **Study characteristics** | | |
| Study design, indication | Multicentre, blinded RCT of patients with ventral hernia | Registry-based, prospective, single-blinded RCT of patients with ventral hernia |
| Country | USA | USA |
| Funding/Sponsor | Investigator-initiated grant from Intuitive Surgical; grants/payments from C-SATS and Activ Surgical reported by 1 author | Intuitive Surgical (IUSI1709AP) |
| Intervention (IG) \| Product | Robotic ventral hernia repair \| NR | Robotic ventral hernia repair \| DaVinci Si or Xi |
| Comparator (CG) | Laparoscopic ventral hernia repair | Laparoscopic ventral hernia repair |
| Experience of surgeon(s); time period | Each centre completed at least 50 standardised repairs as a ramp-up period; 50 cases were selected to ensure operating room staff and surgeons were optimised and to mitigate any possible effect of a learning curve of the standardised repair technique used;  April 2018 – February 2019 (randomisation) | Training in advanced laparoscopy and complex abdominal wall reconstruction; robotic training and credentialing that was in line with requirements defined by Intuitive Surgical and our department of General Surgery;  September 2017 to January 2020 (enrollment) |
| Number of randomised patients | 124; IG: 65; CG: 59 | 81; IG: 39; CG: 39^^[[8]](#footnote-8)^^ (2021)^^[[9]](#footnote-9)^^ |
| Inclusion criteria | - adult patients undergoing elective minimally invasive ventral hernia repair with a defect less than 12 cm wide, and - likely able to tolerate pneumoperitoneum | - ≥18 yrs - presenting in the elective setting with primary or incisional midline ventral hernias of an anticipated width of 7 cm or less who were candidates for minimally invasive hernia repair |
| Primary/secondary endpoints | - clinical outcomes: wound complication, hernia recurrence, port site hernia, readmission, reoperation - patient-reported outcomes: functional status, pain, satisfaction | - primary: pain on the first postoperative day and 1, 7, 30, and 365 days after surgery - secondary: measured preoperatively, at a mean (SD) of 30 (15) days and a mean (SD) of 12 (3) months, included pain as measured by the Patient-Reported Outcomes Measurement Information System (PROMIS) Pain Intensity short form 3a and abdominal-wall–specific QoL using the hernia-specific QoL (HerQLes) survey; operating room time, PACU opioid consumption measured in morphine equivalents, rates of same-day discharge, hospital LOS, as well as surgical site infection, surgical site occurrence, surgical site occurrence requiring a procedural intervention, ventral hernia recurrence, and cost. |
| Follow-up (months) | 12-months postoperative | 30-days postoperative (2021)  12 months postoperative (2022) |
| Drop-outs (n (%)) | IG: 5 (8%); CG: 6 (10%) | After allocation (2021):  IG: 3 (7.7); CG: 3 (7.7)  By 12 months after surgery (2022):  NR |
| **Patient characteristics** | | |
| Age of patients (yrs., mean) | IG: 50.1; CG: 48.0 | *IG vs CG; median (IQR); p-value*  56 (50-70) vs 55 (49-60); NS |
| Sex (% female) | IG: 74%; CG: 63% | IG: 41%; CG: 58%; p=NS |
| BMI (kg/m², mean) | IG: 32.4; CG: 31.8 | *median (IQR); p-value*  IG: 35 (31-39); CG: 31 (27-36); **p=0.02** |
| Clinical classification | ASA (IG vs CG) p=NS  I: 8% vs 8%  II: 57% vs 63%  III: 35% vs 29% | ASA (IG vs CG; n (%) p=NS  I: 1 (3) vs 1 (3)  II: 7 (18) vs 2 (19)  III: 29 (74) vs 27 (75)  IV: 2 (6) vs 1 (3) |
| **Patient-relevant outcomes** | | |
| Survival (overall and disease-specific or disease-free) | NR | NR |
| Recurrence (local, regional or distant) | Hernia recurrence: 4 (7%) vs 5 (9%); NS; relative risk (95% CI): 0.68 (0.17 to 2.68) | *IG vs CG; data captured; n/N (%); p-value*  12-months postoperative:  Hernia recurrence at 1 y: 13/38 (34) vs 6/33 (18); 71/75 (95); NS  Clinical recurrence at 1 y: 5/20 (25) vs 0/17 (NR); 37/75 (49); **p=0.03**  Composite recurrence at 1 y: 9/38 (24) vs 2/33 (6); 71/75 (95); **p=0.04** (2022) |
| Quality of life (e.g. measured by EQ-5D or SF-36) | NR | *Median (IQR); p-value*  **Measured by Hernia-specific quality of life Survey**  30-days postoperative: IG: 67 (45-79); CG: 75 (41 to 81); NS (2021)  *n (95%CI); p-value*  1-y postoperative: IG: 92 (82-100); CG: 77 (49-93); **p=0.04** (2022) |
| Time to resume work/daily activities | NR | NR |
| Patient satisfaction | *IG vs CG; median (interquartile range); p-value; mean difference (95% CI)*  **Visual analogue scale satisfaction at 1-y:**  10.0 (8.0, 10.0) vs 10.0 (7.5, 10.0); NS; 0.3 (-0.7 to 1.3)  *Median (interquartile range); p-value; mean difference (95% CI)*  **Cosmetic satisfaction at 1-y:**  10.0 (5.0, 10.0) vs 10.0 (6.5, 10.0); NS; -0.2 (-1.4 to 1.0) | NR |
| **Safety-related outcomes** | | |
| Intraoperative complications (e.g. air-leakage) | NR | *IG vs CG; n (%); p-value*  **Intraoperative complications (2021):** 2 (6) vs 2 (6); NR  Bowel serosal injury: 1 (3) vs 2 (6); NS  Liver injury: 1 (3) vs 0; NS |
| Postoperative complications (e.g. infections) | *IG vs CG; n (%); p-value; relative risk (95% CI)*  **Wound complication:**  9 (15%) vs 8 (15%); NS; 0.93 (0.32 to 2.74) | *IG vs CG; n (%); p-value*  **Postoperative complications (2021):** 2 (6) vs 3 (8); NS  Pulmonary embolism 1 (3) vs 0; NS  SSO: 0 vs 1 (3); NS  Readmission: 1 (3) vs 1 (3); NS |
| Reoperations/additional surgeries | *IG vs CG; n (%); p-value; mean difference (95% CI)*  **Reoperation:**  0 vs 5 (9%); **p=0.020**; NR | *IG vs CG; n (%); p-value*  **Reoperation**: 0 vs 1 (3); NS (2021)  **Reoperation:** 3 vs 4 (NR); NS (2022) |
| Conversion | NR | *IG vs CG; n (5); p-value*  **Conversion to laparoscopy**: 2 (6) vs NA; NA (2021)  **Conversion to robotic repair:** NA vs 0; NA (2021) |
| **Perioperative events & resource use** | | |
| Blood loss (in ml) | NR | NR |
| Operation time in min. | NR | *IG vs CG; median (IQR); p-value*  146 (123-192) vs 94 (69 -116); **p<0.001** (2021) |
| Transfusions | NR | NR |
| Drain duration (days) | NR | NR |
| Length of hospital stay (days) | NR | *Median (IQR); p-value*  IG: 25 (10 to 30); CG: 10 (8 to 31); NS (2021) |

| **Gallbladder/Liver/Spleen – Hepatectomy** | |
| --- | --- |
| **Author, year** | **Li et al. 2022** |
| **Study characteristics** | |
| Study design, indication | Randomised controlled trial of patients with synchronous colorectal liver metastases |
| Country | China |
| Funding/Sponsor | NR |
| Intervention (IG) \| Product | Robot-assisted laparoscopic hepatectomy \| da Vinci |
| Comparator (CG) | Laparoscopic hepatectomy |
| Experience of surgeon(s); time period | NR; May 2015 to June 2018 (selection) |
| Number of randomised patients | 122; IG: 61; CG: 61 |
| Inclusion criteria | - Patients with synchronous colorectal liver metastases confirmed by clinicopathological diagnosis - treated with radical resection of colorectal cancer, no tumour residue was found - no large blood vessel infiltration, hepatic vein, or portal vein tumour thrombus was found by imaging examination - Child-Pugh liver function class was A or B - No severe organ dysfunction was observed |
| Primary/secondary endpoints | - clinical manifestations (operation time, intraoperative blood transfusion, intraoperative blood loss, average intraoperative blood transfusion, hepatic porta occlusion time - Stress response indicators - Energy metabolism - Complications - Survival |
| Follow-up (months) | 1x/month within the first yr  1x/3 months within the second yr  1x/6 months in the third yr |
| Drop-outs (n (%)) | 3 yrs after surgery  IG: 30 (49.2%); CG: 35 (57.4%); p=NS |
| **Patient characteristics** | |
| Age of patients (yrs., mean) | *IG vs CG; mean (±SD); p-value*  57.13 (± 5.86) vs 57.51 (± 6.27); NS |
| Sex (% female) | IG: 27.9%; CG: 37.7% |
| BMI (kg/m², mean) | *IG vs CG; mean (±SD); p-value*  23.45 ± 2.32 vs 23.59 ± 2.22; NS |
| Clinical classification | *IG vs CG; n (%); p-value*  ASA  1-2: 49 (80.33) vs 44 (72.13); NS  3: 12 (19.67) vs 17 (27.87); NS |
| **Patient-relevant outcomes** | |
| Survival (overall and disease-specific or disease-free) | *IG vs CG; n (%); p-value*  **At 1-yr follow-up:**  52 (85.25) vs 48 (78.69); NS  **At 2-yrs follow-up:**  43 (70.49) vs 40 (65.57); NS  **At 3-yrs follow-up:**  31 (50.82) vs 26 (42.62); NS |
| Recurrence (local, regional or distant) | NR |
| Quality of life (e.g. measured by EQ-5D or SF-36) | NR |
| Time to resume work/daily activities | NR |
| Patient satisfaction | NR |
| **Safety-related outcomes** | |
| Intraoperative complications (e.g. air-leakage) | NR |
| Postoperative complications (e.g. infections) | *IG vs CG; n (%); p-value*  **Total complications**: 2 (3.3) vs 8 (13.1); **p=0.048**  Intestinal obstruction: 1 (1.6) vs 2 (3.3); NR  Bile leakage: 0 (0.0) vs 2 (3.3); NR  Pleural effusion: 1 (1.6) vs 2 (3.3); NR  Abdominal haemorrhage: 0 (0.0) vs 1 (1.6); NR  Incision infection: 0 (0.0) vs 1 (1.6; NR) |
| Reoperations/additional surgeries | NR |
| Conversion | NR |
| **Perioperative events & resource use** | |
| Blood loss (in ml) | *IG vs CG; mean (±SD); p-value*  203.11 (± 10.98) vs 356.00 (± 32.00); **p<0.001** |
| Operation time in min. | *IG vs CG; mean (±SD); p-value*  156.34 (± 15.97) vs 184.18 (± 18.03); **p<0.001** |
| Transfusions | *IG vs CG; mean (±SD); p-value*  608.31 (± 117.08) vs 656.21 (± 103.75); **p=0.018** |
| Drain duration (days) | If no biliary leakage or bleeding was found, the abdominal drainage tube was placed and the operating table was restored to a horizontal position before the abdominal cavity was closed until the end of the operation. (duration NR) |
| Length of hospital stay (days) | NR |

*Abbreviations: ASA = American Society of Anesthesiologists, BMI = body mass index, CG = control group, CI = confidence interval, CRM = circumferential resection margin, d = day, EORTC QLQ-C30 = European Organization for the Research and Treatment of Cancer Quality of Life Questionnaire, EQ-5D = EuroQol 5 Dimension 5 Level, FU = follow-up, GERD = gastroesophageal reflux disease, GORD HRQOL = Gastro-oesophageal Reflux Health-Related Quality of Life scale, GORD = gastro-oesophageal reflux disease, GSRS = Gastrointestinal Symptom Rating Scale, HR = hazard ratio, ICU = intensive care unit, IG = intervention group, IQR = interquartile range, LN = lymph node, M = median, MD = mean difference, min = minutes, mL = millilitres, n = number of patients, NA = not applicable, NE = not evaluable, NR = not reported, NS = not significant, NSCLC = non-small cell lung cancer, PPI = proton pump inhibitor, PSQ = photograph series questionnaire, pts = patients, QoL = Quality of Life, QOLRAD = Quality of Life in Reflux and Dyspepsia, R = range, RATS = Robot-assisted thoracic surgery, RCT = randomised controlled trial, SD = standard deviation, SF-36 = 36-Item Short Form Health Survey, SIRC = single-incision robotic cholecystectomy, TNM = tumour (T), node (N), and metastasis (M), USA = United States of America, VAS = visual analogue scale, VATS = Video-assisted thoracic surgery, vs = versus, wk = week, wks = weeks, y = year, yrs = years, Ø = mean.*

1. Discrepancies could be observed in Jin 2022 and Jin 2023 regarding the randomised patients of the control group. [↑](#footnote-ref-1)
2. Discrepancies in patient characteristics between Table 1 in the publication and Table S1 in the Supplements could be observed. Data extracted from Supplements. [↑](#footnote-ref-2)
3. Discrepancies in postoperative complications between Table 1 in the publication and Table S1 in the Supplements could be observed. Data extracted from Supplements. [↑](#footnote-ref-3)
4. Patient characteristics taken from preciously published study (Müller-Stich 2007). [↑](#footnote-ref-4)
5. Taken from Müller-Stich 2007. [↑](#footnote-ref-5)
6. Death until 90 days after the procedure or during postoperative hospital stay [↑](#footnote-ref-6)
7. Intraoperative hemorrhage more than 100 ml at one time. [↑](#footnote-ref-7)
8. There is an error in the CONSORT flow diagram in the study as 39+39=78. [↑](#footnote-ref-8)
9. Assumed Data in Petro et al. 2021 [↑](#footnote-ref-9)
