# Supplementary material for: Robot-assisted surgery in thoracic and visceral indications: an updated systematic review
Source: Surg Endosc. 2024 Feb 2;38(3):1139–50. doi: 10.1007/s00464-023-10670-1 (PMC10881599; doi:10.1007/s00464-023-10670-1)
Supplement: Supplementary file 2 — Supplementary file2 (DOCX 30 kb) [file 464_2023_10670_MOESM2_ESM.docx]

## **Quality appraisal of the randomised controlled trials using the “Cochrane Collaboration Tool 1”**

Table A6: Risk of bias – study level (RCTs)

| **Trial** | **Adequate generation  of randomization  sequence** | **Adequate allocation concealment** | **Blinding** | | | **Selective outcome  reporting unlikely** | **No other aspects increasing risk of bias** | **Risk of bias – study  level** |
| --- | --- | --- | --- | --- | --- | --- | --- | --- |
|  |  |  | **Patient** | **Treating person^^[[1]](#footnote-1)^^** | |  |  |  |
| **Jin 2022** | **Y^^[[2]](#footnote-2)^^** | **Y^^[[3]](#footnote-3)^^** | **U****^^[[4]](#footnote-4)^^** | | **U^4^** | **U****^^[[5]](#footnote-5)^^** | **Y** | **SC** |
| **Huang 2021** | **Y^^[[6]](#footnote-6)^^** | **N^^[[7]](#footnote-7)^^** | **N****^^[[8]](#footnote-8)^^** | | **N^8^** | **U^5^** | **N****^^[[9]](#footnote-9)^^** | **H** |
| **Terra 2022** | **Y^^[[10]](#footnote-10)^^** | **N^^[[11]](#footnote-11)^^** | **N****^^[[12]](#footnote-12)^^** | | **N^12^** | **N^^[[13]](#footnote-13)^^** | **N^^[[14]](#footnote-14)^^** | **H** |
| **Lang 2022** | **U^4^** | **U^4^** | **U^4^** | **U^4^** | | **Y****^^[[15]](#footnote-15)^^** | **N^^[[16]](#footnote-16)^^** | **H** |
| **Veronesi 2021** | **Y^^[[17]](#footnote-17)^^** | **U^4^** | **U^4^** | **U^4^** | | **N^^[[18]](#footnote-18)^^** | **N^^[[19]](#footnote-19)^^** | **H** |
| **de Groot 2020** | **U^4^** | **U^4^** | **U^4^** | **U^4^** | | **Y^^[[20]](#footnote-20)^^** | **N^^[[21]](#footnote-21)^^** | **H** |
| **Yang 2022** | **N^^[[22]](#footnote-22)^^** | **Y^^[[23]](#footnote-23)^^** | **N****^^[[24]](#footnote-24)^^** | **N^24^** | | **N^^[[25]](#footnote-25)^^** | **Y** | **H** |
| **Lu 2021** | **Y^^[[26]](#footnote-26)^^** | **U^4^** | **N^^[[27]](#footnote-27)^^** | **N^^[[28]](#footnote-28)^^** | | **Y^18^** | **Y** | **SC^^[[29]](#footnote-29)^^** |
| **Ojima 2021** | **Y^^[[30]](#footnote-30)^^** | **U^4^** | **U^4^** | **N^^[[31]](#footnote-31)^^** | | **Y^18^** | **N^^[[32]](#footnote-32)^^** | **H** |
| **Ribeiro 2022** | **Y^^[[33]](#footnote-33)^^** | **U^4^** | **U^4^** | **U^4^** | | **Y** | **Y** | **SC** |
| **Fleming 2022** | **Y^^[[34]](#footnote-34)^^** | **U^4^** | **Y^^[[35]](#footnote-35)^^** | **Y^^[[36]](#footnote-36)^^** | | **U^5^** | **N****^^[[37]](#footnote-37)^^** | **H** |
| **Park 2019** | **Y****^^[[38]](#footnote-38)^^** | **Y^38^** | **N^^[[39]](#footnote-39)^^** | **Y^^[[40]](#footnote-40)^^** | | **Y^18^** | **N^^[[41]](#footnote-41)^^** | **SC^^[[42]](#footnote-42)^^** |
| **Feng 2022a** | **Y^^[[43]](#footnote-43)^^** | **Y^^[[44]](#footnote-44)^^** | **N^^[[45]](#footnote-45)^^** | **Y^^[[46]](#footnote-46)^^** | | **Y^18^** | **Y** | **L** |
| **Feng 2022b** | **Y^^[[47]](#footnote-47)^^** | **N^^[[48]](#footnote-48)^^** | **N^^[[49]](#footnote-49)^^** | **U^^[[50]](#footnote-50)^^** | | **N^^[[51]](#footnote-51)^^** | **Y** | **H** |
| **Dhanani 2021** | **Y^^[[52]](#footnote-52)^^** | **Y^^[[53]](#footnote-53)^^** | **Y****^^[[54]](#footnote-54)^^** | **Y^59^** | | **U^5^** | **N^^[[55]](#footnote-55)^^** | **SC^^[[56]](#footnote-56)^^** |
| **Costa 2023** | **Y^^[[57]](#footnote-57)^^** | **Y^^[[58]](#footnote-58)^^** | **Y** | **N^^[[59]](#footnote-59)^^** | | **Y^18^** | **N^^[[60]](#footnote-60)^^** | **H^^[[61]](#footnote-61)^^** |
| **Olavarria 2020** | **Y^^[[62]](#footnote-62)^^** | **Y^^[[63]](#footnote-63)^^** | **Y^^[[64]](#footnote-64)^^** | **Y^^[[65]](#footnote-65)^^** | | **Y^18^** | **Y** | **L** |
| **Petro 2021** | **Y****^^[[66]](#footnote-66)^^** | **Y^71^** | **Y^^[[67]](#footnote-67)^^** | **N^^[[68]](#footnote-68)^^** | | **Y^^[[69]](#footnote-69)^^** | **Y** | **SC^^[[70]](#footnote-70)^^** |
| **Prabhu 2020** | **Y^^[[71]](#footnote-71)^^** | **U^4^** | **Y^^[[72]](#footnote-72)^^** | **N^^[[73]](#footnote-73)^^** | | **N^^[[74]](#footnote-74)^^** | **N^^[[75]](#footnote-75)^^** | **H** |
| **Li 2022** | **Y^^[[76]](#footnote-76)^^** | **U^4^** | **N^^[[77]](#footnote-77)^^** | **U^4^** | | **Y** | **N^^[[78]](#footnote-78)^^** | **H** |

*Abbreviations: Y= yes, N= no, U= unclear, H= high SC= some concerns, L= low*

1. Since it is impractical for the surgeon to be blinded, we refer here to other healthcare professionals involved in patient care [↑](#footnote-ref-1)
2. “Randomization was conducted with a computer-generated random numbers table.” [↑](#footnote-ref-2)
3. “Assignments were sealed in opaque envelopes, which were opened by the surgeons at the time of the operation.” [↑](#footnote-ref-3)
4. No information given. [↑](#footnote-ref-4)
5. No protocol available. [↑](#footnote-ref-5)
6. “Following a list of randomization number generated in the trial statistician’s computer with stratification for the participating center, the subjects enrolled in present study were randomly and equally assigned.” [↑](#footnote-ref-6)
7. „The allocation was done by telephone by the trial coordinator.” [↑](#footnote-ref-7)
8. “Neither subjects nor any investigators were masked to treatment allocation.” [↑](#footnote-ref-8)
9. No information on power calculation and experience of surgeons given. [↑](#footnote-ref-9)
10. “The research center defined the allocation of the patients using a website software … and used block randomization.” [↑](#footnote-ref-10)
11. „Randomization was not blinded.” Patients were randomised only after having their surgery scheduled, ensuring allocation concealment [↑](#footnote-ref-11)
12. „Randomization was not blinded.” [↑](#footnote-ref-12)
13. Not all predefined outcomes reported, e.g. quality of life. [↑](#footnote-ref-13)
14. No information of experience of surgeons. Sample size might have impacted statistical power. [↑](#footnote-ref-14)
15. Study protocol available. [↑](#footnote-ref-15)
16. „No power calculation was performed.“ [↑](#footnote-ref-16)
17. “Randomization was performed through a dedicated Internet based system with a balance software for center stratification.” [↑](#footnote-ref-17)
18. Secondary outcome data on QoL and recurrence were not reported. [↑](#footnote-ref-18)
19. „The analysis did show adequate statistical power with regard to secondary (not primary) outcomes.” According to the power calculation, “a sample size of 300 subjects was initially calculated.” [↑](#footnote-ref-19)
20. Protocol available. Short-time results published. [↑](#footnote-ref-20)
21. “The number of patients was powered for short-term postoperative outcomes and not specifically for long-term results.” [↑](#footnote-ref-21)
22. “Eligible patients were randomized by the central study coordinator.” [↑](#footnote-ref-22)
23. „Concealment of allocation was performed using computer generated random numbers and further stratified.” [↑](#footnote-ref-23)
24. “There was no blinding for the patient and operator due to practical difficulties.” [↑](#footnote-ref-24)
25. Protocol available. However, mortality is stated but not the overall survival like mentioned in the methods. [↑](#footnote-ref-25)
26. “The SAS 9.2 program was used to generate serial numbers.” [↑](#footnote-ref-26)
27. „The study was not blinded after randomization.” [↑](#footnote-ref-27)
28. „The study was not blinded after randomization.” [↑](#footnote-ref-28)
29. Some concerns as domain blinding not fulfilled and no information given regarding allocation concealment. [↑](#footnote-ref-29)
30. „The minimization method with a random component was used.” [↑](#footnote-ref-30)
31. “Blinding was not applied regarding postoperative management of the patients.” [↑](#footnote-ref-31)
32. No information on experience of surgeons. [↑](#footnote-ref-32)
33. “Participants were assigned by computer-generated simple randomization … using the block randomization method.” [↑](#footnote-ref-33)
34. As stated in a previously published study: “Patients were randomized using a computer-generated randomization code.[[69](#_ENREF_69)] [↑](#footnote-ref-34)
35. “The study was carried out under double-blind conditions.” [[69](#_ENREF_69)] [↑](#footnote-ref-35)
36. “The study was carried out under double-blind conditions.” [[69](#_ENREF_69)] [↑](#footnote-ref-36)
37. No information on the experience of surgeons given. No power calculation. [↑](#footnote-ref-37)
38. “Consenting patients were randomly allocated […] according to a computer-generated random sequence kept concealed by an independent clinical trial coordinator.” [↑](#footnote-ref-38)
39. “Patients […] could not be masked to treatment assignments.” [↑](#footnote-ref-39)
40. “Clinicians could not be masked to treatment assignments. However, during the follow-up period, radiologists and pathologists were masked to the procedural allocation.” [↑](#footnote-ref-40)
41. “The sample size calculation of our trial was based on short-term outcomes such as hospital stay, so our long-term oncological data were inconclusive. Admittedly, the sample size of this study was not adequate.” [↑](#footnote-ref-41)
42. Some concerns as no patient blinding was done and only short-term outcomes were conclusive. [↑](#footnote-ref-42)
43. „A simple randomization method was used with a computer‐generated random number sequence in this trial.” [↑](#footnote-ref-43)
44. „An independent statistician made and kept the envelopes containing group numbers to conceal the sequence. After eligibility and informed consent, one envelope was opened by the principal investigator of this trial to decide the allocation for each patient.” [↑](#footnote-ref-44)
45. „No blinding to treatment allocation was incorporated in this trial.” [↑](#footnote-ref-45)
46. „The outcomes were evaluated and recorded by two blinded assessors according to medical documents without information on the grouping allocation.“ [↑](#footnote-ref-46)
47. „An online central randomization system was used for allocation. Randomisation was stratified according to [defined] factors.” [↑](#footnote-ref-47)
48. „The principal investigator of each participating centre logged onto the system website, obtained the random allocation, and informed the patient.” [↑](#footnote-ref-48)
49. „The investigators and patients were not blinded to the treatment allocation.” [↑](#footnote-ref-49)
50. „The investigators and patients were not blinded to the treatment allocation. However, the senior pathologists of each participating centre were masked to the assessment of pathological outcomes.” [↑](#footnote-ref-50)
51. Protocol available. However, outcomes on survival and quality of life (follow-up) are not reported. [↑](#footnote-ref-51)
52. „Patients were randomized by computer-generated, variable block in a 1:1 ratio, stratified by surgeon.” [↑](#footnote-ref-52)
53. „Treatment allocation was determined through opening of sequentially numbered, opaque, sealed envelopes.” [↑](#footnote-ref-53)
54. „The operating surgeons and research coordinators who determined treatment allocation could not be blinded given the nature of the intervention. However, the patients and post-operative outcome assessors were blinded to the patients’ allocation group.” [↑](#footnote-ref-54)
55. „Given our results and assuming true effect size is 50% lower (4.5% vs 0.5% reoperation rate), 476 patients would be needed for an appropriately powered study to detect a true difference.” [↑](#footnote-ref-55)
56. Some concerns as only 124 patients were included in the trial (not 476 patients). [↑](#footnote-ref-56)
57. “An independent coordinator nurse using the Microsoft Excel random number generation function performed a randomization [↑](#footnote-ref-57)
58. “The number generated was kept blinded to the patient in a sequentially numbered opaque sealed envelope.” [↑](#footnote-ref-58)
59. Single-blinded trial. [↑](#footnote-ref-59)
60. There was a “lack of reasonable sample size estimation, and each outcome followed a per-protocol analysis.” [↑](#footnote-ref-60)
61. High risk of bias as domain “blinding of treating person” not fulfilled, lack of reasonable sample size estimation, and each outcome followed a per-protocol analysis. [↑](#footnote-ref-61)
62. „Randomisation… by using a computer generated variable block randomization schema stratified by surgeon.” [↑](#footnote-ref-62)
63. „Surgeons contacted the research assistant, who determined the treatment allocation through opening of sequentially numbered opaque sealed envelopes.” [↑](#footnote-ref-63)
64. „The patient and the rest of the research team, including postoperative outcome assessors, were all blinded to the patients’ allocation group.” [↑](#footnote-ref-64)
65. “Operating surgeons and the research coordinator who determined the randomization allocation could not be blinded.” „The patient and the rest of the research team, including postoperative outcome assessors, were all blinded to the patients’ allocation group.” [↑](#footnote-ref-65)
66. „A concealed randomization scheme was performed by using a random number of blocks with a 1:1 ratio of assigning patients to each arm.” [↑](#footnote-ref-66)
67. „Patients were blinded to the operative approach throughout the study.“ [↑](#footnote-ref-67)
68. Single-blinded study. [↑](#footnote-ref-68)
69. Protocol available. [↑](#footnote-ref-69)
70. Some concerns as only domain “blinding of treating person” is not fulfilled. [↑](#footnote-ref-70)
71. „The randomization was performed using a random number of blocks with 1:1 ratio of assigning patients to each group.” [↑](#footnote-ref-71)
72. „Patients were blinded to their interventions.” [↑](#footnote-ref-72)
73. Single-blinded study. [↑](#footnote-ref-73)
74. Protocol available. However, not all outcomes reported (i.e. hernia recurrence rates, cosmetic results). [↑](#footnote-ref-74)
75. “There was essentially no precedent on which to perform a power calculation as robotic adoption was in its infancy for repair of inguinal hernia. Thus, this study was designed as a pilot study.” [↑](#footnote-ref-75)
76. Patients were selected “according to random number table method”. [↑](#footnote-ref-76)
77. Patients were fully informed. [↑](#footnote-ref-77)
78. No power calculation. No information on experience of surgeons. [↑](#footnote-ref-78)
