# Supplementary material for: Robot-assisted surgery in thoracic and visceral indications: an updated systematic review
Source: Surg Endosc. 2024 Feb 2;38(3):1139–50. doi: 10.1007/s00464-023-10670-1 (PMC10881599; doi:10.1007/s00464-023-10670-1)
Supplement: Supplementary file 3 — Supplementary file3 (DOCX 170 kb) [file 464_2023_10670_MOESM3_ESM.docx]

**Certainty of evidence**

Table A7: Summary of Findings of robot-assisted surgery in thoracic and visceral indications

| Outcomes | Indication | Comparison | Impact | N studies (pts IG vs CG) | Certainty of Evidence |
| --- | --- | --- | --- | --- | --- |
| **Effectiveness- Patient-relevant outcomes** | | | | | |
| **Survival** | Lung | Video-assisted lobectomy/ video-assisted thoracic surgery | 1 RCT: **Deaths 48-wks postoperatively:** IG: 7; CG: 14 (2023)  1 RCT: **Mortality within 90 days after surgery:** IG: 1 (2.7); CG: 1 (2.5); p=NS | 2 RCTs [[37](#_ENREF_37), [39](#_ENREF_39)] 221 vs 222) | Very low ⨁**◯◯◯** |
|  |  | Open-surgery | 1 RCT: *IG vs CG; %; p-value*  **Disease-free survival:**  1 yr: 90.4 vs 86.0; NS  2 yrs: 76.4 vs 74.2; NS | 1 RCT [[32](#_ENREF_32)] (137 vs 133) | Low ⨁⨁**◯◯** |
|  | Oesophagus | Conventional laparoscopic fundoplication/ conventional minimally invasive oesophagectomy | 1 RCT: *IG vs CG; n (%);* p=NS  **In-hospital mortality:** 0 (0) vs 0 (0)  30-d mortality: 0 (0) vs 1 (0.6)  90-d mortality: 1 (0.6) vs 1 (0.6) | 1 RCT [[42](#_ENREF_42)] (183 vs 179) | Low ⨁⨁**◯◯** |
|  |  | Open-surgery | 1 RCT: *IG vs CG; median in months (range); rate (95% CI); p=NS*  **Overall survival:** 35 (1–60); 41% (95% CI 27–55) vs 41 (2–60); 40% (95% CI 26–53)  **Disease-free survival:** 28 (0–56); 42% (95% CI 28–55) vs 37 months (3–56); 43% (95% CI 29–57) | 1 RCT [[43](#_ENREF_43)] (56 vs 56) | Low ⨁⨁**◯◯** |
|  | Stomach | Laparoscopic (distal) gastrectomy | 1 RCT: *IG vs CG; n (%); p-value*  **In-hospital mortality within 30 days postoperative:** 0 (0) vs 0 (0); NA  1 RCT: *IG vs CG; n (%); p-value* (per-protocol analysis)  **Mortality:** IG: 0; CG: 0; p=NS | 2 RCTs [[44](#_ENREF_44), [45](#_ENREF_45)] (269 vs 272) | Low ⨁⨁**◯◯** |
|  |  | Open surgery | 1 RCT: **Mortality^[[1]](#footnote-1)^:**  IG: 0; CG: 0; p=NR | 1 RCT [[46](#_ENREF_46)] (33 vs 32) | Moderate ⨁⨁⨁**◯** |
|  | Bowel | Laparoscopic surgery/ laparoscopic ventral mesh rectopexy | 1 RCT: *IG vs CG; mean (%) (95% CI); p-value*  **Disease-free survival:**  3 yrs after surgery: 88.1 (77.1–99.1) vs 91.1 (81.4–99.9); NS  5 yrs after surgery: 77.4 (60.6–92.1) vs 83.6 (72.1–97.0); NS  **Overall survival:**  3 yrs after surgery: 96.8 (90.6–99.9) vs 94.0 (86.0-99.9); NS  5 yrs after surgery: 91.1 (78.8–99.9) vs 91.0 (81.3–99.9); NS  1 RCT: **Disease‐free survival** (3‐yrs rate of stage I−III pts):  85.3% vs 84.6% (log‐rank NS; HR=0.918; 95% CI = 0.555−1.517); NS  **Overall survival** (3‐yrs rate of all pts):  91.1% vs 90.4% (log‐rank NS; HR=0.912; 95% CI = 0.490−1.697); NS | 2 RCTs [[47](#_ENREF_47), [49](#_ENREF_49)] (209 vs 209) | Moderate ⨁⨁⨁**◯** |
|  | Gallbladder/Liver/Spleen | Laparoscopic ventral/incisional hernia repair/ laparoscopic transabdominal preperitoneal repair/ laparoscopic repair/ laparoscopic hepatectomy | 1 RCT: *IG vs CG; n (%); p-value*  **Mortality** (short-term, within 7 days): 0 vs 1 (5); NS  1 RCT: *IG vs CG; n (%); p-value*  **At 1-yr follow-up:**  52 (85.25) vs 48 (78.69); NS  **At 2-yrs follow-up:**  43 (70.49) vs 40 (65.57); NS  **At 3-yrs follow-up:**  31 (50.82) vs 26 (42.62); NS | 2 RCTs [[54](#_ENREF_54), [60](#_ENREF_60)] (81 vs 81) | Very Low ⨁◯◯◯ |
| **Recurrence** | Lung | Video-assisted lobectomy/ video-assisted thoracic surgery | 1 RCT: **Recurrence 48-wks postoperatively:** IG: 6; CG:5 (2023) | 1 RCT [[37](#_ENREF_37)] (181 vs 182) | Low ⨁⨁**◯◯** |
|  |  | Open surgery | NR | | |
|  | Oesophagus | Conventional laparoscopic fundoplication/ conventional minimally invasive oesophagectomy | 1 RCT: *IG vs CG; n (%); p=NR*  **Failure of treatment:**  Oesophagitis ≥ LA-B: 1 (8) vs 1 (8)  GSRS reflux score ≥ 3: 3 (25) vs 2 (17)  Daily PPI for reflux: 4 (31) vs 4 (33)  Dysphagia combined with reflux score ≥ 2: 1 (8) vs 1 (8) | 1 RCT [[41](#_ENREF_41)] (20 vs 20) | Low ⨁⨁**◯◯** |
|  |  | Open surgery | 1 RCT: *IG vs CG; n (%); p=NS*  **Overall recurrence disease:** 28 (56) vs 29 (54) | 1 RCT [[43](#_ENREF_43)] (56 vs 56) | Moderate  ⨁⨁⨁**◯** |
|  | Stomach | Laparoscopic (distal)  gastrectomy | NR | | |
|  |  | Open surgery | NR | | |
|  | Bowel | Laparoscopic surgery/ laparoscopic ventral mesh rectopexy | 1 RCT: No port site recurrence was noted with a median follow-up of 49 months  1 RCT: **Recurrence at 3 yrs after surgery** (IG (n=173) vs CG (n=173); difference (95% CI); p-value):  **Locoregional recurrence:** 5 (2.9) vs 9 (5.2); -2.3 (-7.0 to 2.1); NS  **Distant metastases:** 21 (12.1) vs 23 (13.3); -1.2 (-8.3 to 6.0); NS  1 RCT: At 24-month follow-up (2019):  IG: 0 vs CG: 1 (8%); p=NR | 3 RCTs [[47](#_ENREF_47), [49](#_ENREF_49), [52](#_ENREF_52)] (225 vs 223) | Very low ⨁**◯◯◯** |
|  | Gallbladder/Liver/Spleen | Laparoscopic ventral/incisional hernia repair/ laparoscopic transabdominal preperitoneal repair/ laparoscopic repair/ laparoscopic hepatectomy | 1 RCT: Hernia recurrence: 4 (7%) vs 5 (9%); NS; relative risk (95% CI): 0.68 (0.17 to 2.68)  1 RCT: *IG vs CG; data captured; n/N (%); p-value*  12-months postoperative:  **Hernia recurrence at 1 y:** 13/38 (34) vs 6/33 (18); 71/75 (95); NS  **Clinical recurrence at 1 y:** 5/20 (25) vs 0/17 (NR); 37/75 (49); **p=0.03**  **Composite recurrence at 1 y**: 9/38 (24) vs 2/33 (6); 71/75 (95); **p=0.04** (2022)  1 RCT: IG: 2 (11.1); CG: 3 (15.75) (in 24-month-follow-up)  1 RCT: *IG vs CG; n;* p=NS  NR (2020)  Inguinal hernia recurrence:  2 yrs after surgery: 1 vs 1 (2023)  1 RCT: IG: 0 (0%); CG: 0 (0%); p=NS | 5 RCTs [[54-59](#_ENREF_54)] (237 vs 231) | Very Low ⨁**◯◯◯** |
| **Quality of Life** | Lung | Video-assisted lobectomy/ video-assisted thoracic surgery | 1 RCT: **QoL:**  *Mean difference (95% CI)*  4 wks 0.002 (–0.008~0.012)  24 wks 0.003 (–0.004~0.010)  48 wks 0.004 (–0.002~0.011) | 1 RCT [[37](#_ENREF_37), [40](#_ENREF_40)] (181 vs 182) | Moderate ⨁⨁⨁**◯** |
|  |  | Open surgery | NR | | |
|  | Oesophagus | Conventional laparoscopic fundoplication/ conventional minimally invasive oesophagectomy | 1 RCT: *IG vs CG; mean ± SD (range);* p=NS  **Quality of life in reflux and dyspepsia:**  Emotional distress: 6.4 ± 1.4 (1.2–7.0) vs 6.5 ± 1.6 (1.0–7.0)  Food/drink problems: 6.5 ± 0.9 (3.5–7.0) vs 6.3 ± 1.6 (1.0–7.0)  Physical/social functioning: 6.6 ± 1.0 (2.8–7.0) vs 6.4 ± 1.6 (1.0–7.0)  Sleep disturbance: 6.4 ± 1.3 (2.2–7.0) vs 6.5 ± 1.5 (1.0–7.0)  Vitality: 6.3 ± 1.4 (1.3–7.0) vs 6.3 ± 1.6 (1.0–7.0) | 1 RCT [[41](#_ENREF_41)] (20 vs 20) | Low ⨁⨁**◯◯** |
|  |  | Open surgery | NR | | |
|  | Stomach | Laparoscopic (distal) gastrectomy | NR | | |
|  |  | Open surgery | NR | | |
|  | Bowel | Laparoscopic surgery/ laparoscopic ventral mesh rectopexy | 1 RCT: *IG vs CG; n; mean (SD); difference between means (95% CI); p-value*  **QoL measurements 5 yrs postoperative (2020):**  **CRAIQ-7:** 14; 24.3 (32.0) vs 10; 43.8 (27.1); -20.4 (-43.2 to 2.5); NS  **POPIQ-7:** 13; 9.5 (26.4) vs 10; 26.0 (27.9); -16.1 (-39.7 to 7.5); NS  **UIQ-7:** 14; 25.7 (32.7) vs 10; 33.0 (31.4); -9.4 (-32.3 to 13.6); NS  **PFIQ-7:** 14; 58.8 (82.1) vs 10; 102.7 (69.9); -47.8 (-103.7 to 8.0); NS | 1 RCT [[52](#_ENREF_52)] (16 vs 14) | Low ⨁⨁**◯◯** |
|  | Gallbladder/Liver/Spleen | Laparoscopic ventral/incisional hernia repair/ laparoscopic transabdominal preperitoneal repair/ laparoscopic repair/ laparoscopic hepatectomy | 1 RCT: *n (95%CI); p-value*  **Measured by Hernia-specific quality of life Survey**  1-y postoperative: IG: 92 (82-100); CG: 77 (49-93); **p=0.04** (2022)  1 RCT: *IG vs CG; mean (SD); p-value*  Evaluated with the EORTC QLQ-C30  **2-yrs after surgery:**  Global health: 72.07 (22.67) vs 67.69 (26.32); NS  Functional: 77.27 (19.85) vs 67.19 (21.40); NS  Symptoms: 22.13 (14.72) vs 30 (19.15); NS  1 RCT: *IG vs CG; mean (SD); p-value*  Measured with the SF-36  **30 days after surgery:**  Physical component summary: -1.98 (8.90) vs -0.59 (8.91); NS  Mental component summary: 0.71 (5.84) vs 0.65 (8.29); NS  General Health: 1.55 (8.43) vs -2.31 (12.4); NS  1 RCT: *IG vs CG; mean (SD); p-value*  **2-yrs after surgery:**  Physical component summary: 53.1 (8.1) vs 54.2 (6.1); NS  Mental component summary: 53.9 (6.8) vs 53.4 (5.6); NS | 4 RCTs [[53](#_ENREF_53), [54](#_ENREF_54), [56-59](#_ENREF_56)] (172 vs 172) | Very Low ⨁**◯◯◯** |
| **Safety- Safety-related outcomes** | | | | | |
| **Intraoperative Complications** | Lung | Video-assisted lobectomy/ video-assisted thoracic surgery | 1 RCT: IG: 0; CG: 3; p=NS  (2 arterial lacerations and 1 venous injury) | 1 RCT [[39](#_ENREF_39)] (40 vs 40) | Low ⨁⨁**◯◯** |
|  |  | Open surgery | NR | | |
|  | Oesophagus | Conventional laparoscopic fundoplication/ conventional minimally invasive oesophagectomy | 1 RCT: Conversion to open surgery: **IG:** 7 (3.9%) vs **CG:** 6 (3.4%) | 1 RCT [[42](#_ENREF_42)] (183 vs 179) | Moderate ⨁⨁⨁◯ |
|  |  | Open surgery | NR | | |
|  | Stomach | Laparoscopic (distal) gastrectomy | NR | | |
|  |  | Open surgery | 1 RCT: IG: 0; CG: NR; p=NR | 1 RCT [[46](#_ENREF_46)] (33 vs 32) | Moderate ⨁⨁⨁**◯** |
|  | Bowel | Laparoscopic surgery/ laparoscopic ventral mesh rectopexy | 1 RCT: *IG vs CG; n (%); p-value*  3 (7) vs 4 (5); NS  1 RCT: *IG vs CG; n (%); p-value*  **Pts with any intraoperative complications:** 10 (5.7) vs 16 (9.2); NS  1 RCT: *IG vs CG; n (%); difference (95% CI); p-value*  **Intraoperative complications**: 32 (5.5%) vs 51 (8.7%); –3·3 (–6·3 to –0·3); **p=0.030**  **Significant bleeding:** 16 (2.7%) vs 26 (4.4%); –1·7 (–4·0 to 0·4); NS | 3 RCTs [[48-50](#_ENREF_48)] (837 vs 877) | Very low ⨁**◯◯◯** |
|  | Gallbladder/Liver/Spleen | Laparoscopic ventral/incisional hernia repair/ laparoscopic transabdominal preperitoneal repair/ laparoscopic repair/ laparoscopic hepatectomy | 1 RCT: *IG vs CG; n (%); p-value*  **Intraoperative complications (2021):** 2 (6) vs 2 (6); NR  Bowel serosal injury: 1 (3) vs 2 (6); NS  Liver injury: 1 (3) vs 0; NS | 1 RCT [[53](#_ENREF_53)] (39 vs 39) | Moderate ⨁⨁⨁**◯** |
| **Postoperative Complications** | Lung | Video-assisted lobectomy/ video-assisted thoracic surgery | 1 RCT: *IG vs CG; n (%); p-value*  **Postoperative complications:** 23 (14.6) vs 30 (18.4); NS  **Clavien Dindo I-II:** 18 (11.5) vs 24 (14.7); NS  **Clavien Dindo III-IV:** 5 (3.2) vs 6 (3.7); NS  **Readmission:** 3 (1.9) vs 3 (1.8); NS  1 RCT: *IG vs CG; n (%); p-value*  **Early postoperative complications^[[2]](#footnote-2)^:** 13 (37) vs 9 (24); NS  **Readmissions:** 4 (16) vs 0 (0); NS  **Later Complication:** 5(23) vs 2 (11); NS  1 RCT: *IG vs CG; n (%); p-value*  **Complications within 90 days:** 7 (18.9) vs 14 (35.9); NS  **≥ 3 complications within 90 days:** 7 (18.9) vs 10 (25.6); NS  **Readmissions within 90 days:** 1 (2.7) vs 8 (20.5); **p=0.029** | 3 RCTs [[37-39](#_ENREF_37)] (259 vs 261) | Very low ⨁**◯◯◯** |
|  |  | Open surgery | 1 RCT: *IG vs CG; n (%); p-value*  Prolonged air leak: 6 (7.9) vs 6 (8.3); NS  Bronchopleural fistula: 4 (5.3) vs 1 (1.4); NS  Pneumonia: 3 (3.9) vs 6 (8.3); NS  Hyperpyrexia: 2 (2.6) vs 6 (8.3); NS  Haemorrhage: 2 (2.6) vs 1 (1.4); NS  Recurrent laryngeal nerve injury: 1 (1.3) vs 4 (5.6); NS  Pulmonary embolism: 1 (1.3) vs 0; NS | 1 RCT [[32](#_ENREF_32)] (137 vs 133) | Low ⨁⨁**◯◯** |
|  | Oesophagus | Conventional laparoscopic fundoplication/ conventional minimally invasive oesophagectomy | 1 RCT: *IG vs CG; n (%);* p=NS  **Total complications:** 88 (48.6) vs 74 (41.8)  C-D classification ≥ III: 22 (12.2) vs 18 (10.2)  **Pulmonary complications:** 25 (13.8) vs 26 (14.7)  **Severe cardiac complications:** 2 (1.1) vs 1 (0.6)  **Anastomotic leakage:** 22 (12.2) vs 20 (11.3)  **Vocal cord paralysis:** 59 (32.6) vs 48 (27.1) | 1 RCT [[42](#_ENREF_42)] (183 vs 179) | Moderate ⨁⨁⨁◯ |
|  |  | Open surgery | NR | | |
|  | Stomach | Laparoscopic (distal) gastrectomy | 1 RCT: *IG vs CG; n (%); p-value*  **Overall morbidity:** 13 (9.2) vs 25 (17.6); **p=0.039**  **Surgical morbidity:** 5 (3.5) vs 9 (6.3); NS  **Medical morbidity:** 9 (6.4) vs 20 (14.1); **p=0.033**  **Clavien-Dindo classification:**  I: 0 (0.0) vs 0 (0.0); NS; II: 11 (7.8) vs 22 (15.5); NS; IIIa: 0 (0.0) vs 1 (0.7); NS; IIIb: 1 (0.7) vs 1 (0.7); NS; IV: 1 (0.7) vs 1 (0.7); NS; V: 0 (0.0) vs 0 (0.0); NS  1 RCT: *IG vs CG; n (%); p-value* (per-protocol analysis)  **Overall complications, ≥grade IIb:** 10 (8.8) vs 23 (19.7); **p=0.02**  **Overall complications, ≥grade IIIa:** 6 (5.3) vs 19 (16.2); **p=0.01**  **Surgical complications:**  Anastomotic leakage, ≥grade II: 4 (3.5) vs 5 (4.3); NS  Anastomotic leakage, ≥grade IIIa: 3 (2.7) vs 5 (4.3); NS  Intra-abdominal abscess, ≥grade II: 3 (2.7) 3 (2.6); NS  Intra-abdominal abscess, ≥grade IIIa: 2 (1.8) vs 3 (2.6); NS  **Medical complications:**  Pneumonia, ≥grade II: 1 (0.9) vs 5 (4.3); NS | 2 RCTs [[44](#_ENREF_44), [45](#_ENREF_45)] (269 vs 272) | Low ⨁⨁**◯◯** |
|  |  | Open surgery | 1 RCT: *IG vs CG; n (%); p-value*  **Postoperative complications (0- 30 days postoperative):**  Minor: 4 (13.8) vs 6 (19.4); NS  Major: 4 (13.8) vs 3 (3.2); NS  **Late complications (>30 days postoperative):** 1 (3.4) vs 6 (19.4); NS  **Readmission** (<90 days): IG: 1 (3.4); CG: 4 (12.9); NS | 1 RCT [[46](#_ENREF_46)] (33 vs 32) | Moderate ⨁⨁⨁**◯** |
|  | Bowel | Laparoscopic surgery/ laparoscopic ventral mesh rectopexy | 1 RCT: *IG vs CG; n (%); p-value*  **Postoperative surgical complication:** 7 (16) vs 10 (12); NS  **Anastomotic leak:** 2 (5) vs 3 (4); NS  **Medical complication:** 4 (9) vs 8 (10); NS  **Clavien Dindo:**  0: 35 (81) vs 68 (81); NS; I: 3 (7) vs 3 (4); NS; II: 3 (7) vs 7 (8); NS; III: 2 (5) vs 4 (5); NS; IV: 0 (0) vs 2 (2); NS  1 RCT: *IG vs CG;n (%); p-value*  **Wound infection:** 2 (5.6) vs 2 (5.6); NR  **Anastomosis leakage:** 1 (2.8) vs 0 (0); NR  **Intraabdominal abscess:** 0 (0) vs 1 (2.8); NR  1 RCT: *IG vs CG: n (%); unadjusted difference (95% CI); p-value (within 30 days after surgery)*  **Total 30-day postoperative complication rate (Clavien−Dindo grade II or higher):** 23  1 RCT: *IG vs CG: n (%); difference (95% CI); p-value (within 30 days after surgery)*  **Complications of Clavien–Dindo grade II or higher grade within 30 days after operation:**  95 (16.2) vs 135 (23.1); -6.9 (-11.4 to -2.3); p=0.003  **Readmissions within 30 days after operation:** 17 (2.9) vs 20 (3.4); –0.5 (–2.6 to 1.6); NS | 4 RCTs [[47-50](#_ENREF_47), [57](#_ENREF_57)] (872 vs 913) | Very low ⨁**◯◯◯** |
|  | Gallbladder/Liver/Spleen | Laparoscopic ventral/incisional hernia repair/ laparoscopic transabdominal preperitoneal repair/ laparoscopic repair/ laparoscopic hepatectomy | 1 RCT: *IG vs CG; n (%); p-value; relative risk (95% CI)*  **Wound complication:** 9 (15%) vs 8 (15%); NS; 0.93 (0.32 to 2.74)  1 RCT: *IG vs CG; n (%); p-value*  **Postoperative complications (2021):** 2 (6) vs 3 (8); NS  1 RCT: *IG vs CG; n (%); p-value*  Complications (short-term, within 7 days): 3 (16.7) vs 2 (10.5); NS  1 RCT: *IG vs CG; n (%); p-value*  30-days after surgery: **Adverse Events:** 8 (16.7) vs 5 (9.3); NS  1 RCT: *IG vs CG; n (%); relative rate (95% CI); p-value*  **Readmission:** 1 (2) vs 3 (5); 0.27 (0.03 to 2.43); p=NS  **Emergency room visits:** 7 (11) vs 5 (9); 1.28 (0.43 to 3.75); p=NS  **Wound complication:** 13 (20) vs 11 (19); 1.02 (0.51 to 2.08); p=NS  **Clavien-Dindo complication:** 14 (22) vs 11 (19); 1.10 (0.54 to 2.24); NS  1-2: 14 (22) vs 10 (17); NR; NR  3-5: 0 (0) vs 1 (2); NR; NR  1 RCT: *IG vs CG; n (%); p-value*  **Total complications**: 2 (3.3) vs 8 (13.1); **p=0.048** | 6 RCTs [[53-60](#_ENREF_53)] (298 vs 292) | Very Low ⨁**◯◯◯** |

Abbreviations: CG = control group, CI = confidence interval, IG = intervention group, n/N = number of patients, NR = not reported, NS = not significant, pts = patients, QoL = Quality of Life, RCT(s) = randomized controlled trial(s), SD = standard deviation, SF-36 = 36-Item Short Form Health Survey, vs = versus, yr = year, yrs = years.

1. Death until 90 days after the procedure or during postoperative hospital stay [↑](#footnote-ref-1)
2. Discrepancies in postoperative complications between Table 1 in the publication and Table S1 in the Supplements could be observed. Data extracted from Supplements. [↑](#footnote-ref-2)
