# Supplementary material for: Robot-assisted surgery in thoracic and visceral indications: an updated systematic review
Source: Surg Endosc. 2024 Feb 2;38(3):1139–50. doi: 10.1007/s00464-023-10670-1 (PMC10881599; doi:10.1007/s00464-023-10670-1)
Supplement: Supplementary file 4 — Supplementary file4 (DOCX 31 kb) [file 464_2023_10670_MOESM4_ESM.docx]

**Search Syntax**

**Database: Ovid MEDLINE(R) ALL <1946 to April 18, 2023>**

Search Strategy:

--------------------------------------------------------------------------------

1 exp Robotic Surgical Procedures/ (15400)

2 robot*-assisted*.mp. (19709)

3 (robot* adj5 (surger* or surgical*)).mp. (27135)

4 1 or 2 or 3 (34479)

5 ((pulmonary or lung*) adj5 (segmentectom* or lobectom*)).mp. (5574)

6 ((excis* or resect*) adj5 (lobe* or lung*)).mp. (26301)

7 5 or 6 (30124)

8 4 and 7 (540)

9 limit 8 to clinical trial, all (11)

10 ((randomized controlled trial or controlled clinical trial).pt. or randomized.ab. or placebo.ab. or clinical trials as topic.sh. or randomly.ab. or trial.ti.) not (exp animals/ not humans.sh.) (1396957)

11 8 and 10 (30)

12 9 or 11 (34)

13 limit 12 to (english or german) (33)

14 limit 13 to dt=20180626-20230417 (19)

15 limit 13 to ed=20180626-20230417 (12)

16 14 or 15 (19)

17 exp Mediastinum/su [Surgery] (1016)

18 (mediastin* adj5 (surg* or resect*)).mp. (5255)

19 exp Thymectomy/ (8272)

20 thymectom*.mp. (11057)

21 exp Thymus Gland/su [Surgery] (517)

22 (thymus adj5 (surg* or resect* or excis* or remov*)).mp. (879)

23 17 or 18 or 19 or 20 or 21 or 22 (17421)

24 4 and 23 (383)

25 limit 24 to clinical trial, all (5)

26 10 and 24 (17)

27 25 or 26 (20)

28 limit 27 to (english or german) (19)

29 limit 28 to dt=20180704-20230417 (10)

30 limit 28 to ed=20180704-20230417 (8)

31 29 or 30 (11)

32 exp Gastroesophageal Reflux/ (29103)

33 reflux.mp. (69499)

34 GER.mp. (3725)

35 GERD.mp. (10376)

36 GORD.mp. (909)

37 (plication* or fundic wrap*).mp. (4337)

38 anti-reflux.mp. (1764)

39 anti?reflux.mp. (4842)

40 exp FUNDOPLICATION/ (5080)

41 fundoplication*.mp. (7847)

42 32 or 33 or 34 or 35 or 36 or 37 or 38 or 39 or 40 or 41 (78723)

43 4 and 42 (506)

44 limit 43 to clinical trial, all (18)

45 10 and 43 (34)

46 44 or 45 (41)

47 45 or 46 (41)

48 limit 47 to (english or german) (38)

49 limit 48 to dt=20180801-20230417 (7)

50 limit 48 to ed=20180801-20230417 (6)

51 49 or 50 (8)

52 exp Esophagectomy/ (12349)

53 Oesophagectom*.mp. (1886)

54 Esophagectom*.mp. (16999)

55 ((Trans?hiat* or Trans-hiat*) adj3 (Oesophagectom* or Esophagectom*)).mp. (885)

56 ((oesophag* or esophag*) adj3 (remov* or excis* or resect*)).mp. (7310)

57 52 or 53 or 54 or 55 or 56 (22011)

58 4 and 57 (516)

59 limit 58 to clinical trial, all (18)

60 10 and 58 (55)

61 59 or 60 (59)

62 limit 61 to (english or german) (56)

63 limit 62 to dt=20180808-20230417 (39)

64 limit 62 to ed=20180808-20230417 (31)

65 63 or 64 (40)

66 exp Esophageal Perforation/ (4518)

67 ((oesophag* or esophag* or Heller*) adj3 (repair* or perforat* or myotom*)).mp. (8833)

68 exp Heller Myotomy/ (266)

69 LHM.ti,ab. (383)

70 exp Esophageal Achalasia/ (7667)

71 achalasia*.mp. (9614)

72 ((oesophag* or esophag*) adj3 (swallow* adj3 (disorder* or difficult* or problem* or impair*))).mp. (73)

73 ((oesophag* or esophag*) adj3 dysphagia*).mp. (1632)

74 66 or 67 or 68 or 69 or 70 or 71 or 72 or 73 (19055)

75 4 and 74 (124)

76 limit 75 to clinical trial, all (4)

77 10 and 75 (20)

78 76 or 77 (23)

79 limit 78 to (english or german) (20)

80 limit 79 to dt=20180822-20230417 (6)

81 limit 79 to ed=20180822-20230417 (4)

82 80 or 81 (6)

83 exp Gastrectomy/ (40644)

84 Gastrectom*.mp. (53232)

85 Pylorectom*.mp. (72)

86 ((stomach or pylor*) adj3 (remov* or excis* or resect*)).mp. (3690)

87 83 or 84 or 85 or 86 (55443)

88 4 and 87 (744)

89 limit 88 to clinical trial, all (32)

90 10 and 88 (90)

91 89 or 90 (103)

92 limit 91 to (english or german) (100)

93 limit 92 to dt=20180824-20230417 (45)

94 limit 92 to ed=20180824-20230417 (44)

95 93 or 94 (48)

96 exp Bariatric Surgery/ (32998)

97 bariatric*.mp. (28876)

98 (Gastric adj3 (bypass* or band* or stimul*)).mp. (24245)

99 Roux*.mp. (17459)

100 RYGB.ti,ab. (4111)

101 (sleeve* adj3 gastrectom*).mp. (8481)

102 96 or 97 or 98 or 99 or 100 or 101 (56710)

103 4 and 102 (527)

104 limit 103 to clinical trial, all (8)

105 10 and 103 (32)

106 104 or 105 (35)

107 limit 106 to (english or german) (33)

108 limit 107 to dt=20180830-20230417 (13)

109 limit 107 to ed=20180830-20230417 (16)

110 108 or 109 (17)

111 exp Intestine, Small/ (167611)

112 ((small bowel* or small intestine*) adj3 (remov* or excis* or resect*)).mp. (4527)

113 111 or 112 (169737)

114 4 and 113 (230)

115 limit 114 to clinical trial, all (2)

116 10 and 114 (7)

117 115 or 116 (9)

118 limit 117 to (english or german) (8)

119 limit 118 to dt=20180831-20230417 (2)

120 limit 118 to ed=20180831-20230417 (4)

121 119 or 120 (4)

122 exp Colectomy/ (23301)

123 colectom*.mp. (26877)

124 procto?colectom*.mp. (5407)

125 hemi?colectom*.mp. (5017)

126 sigmoidectom*.mp. (1226)

127 transversectom*.mp. (31)

128 ((colon* or hemi*colon* or sigmoid*) adj3 (remov* or excis* or resect*)).mp. (11062)

129 122 or 123 or 124 or 125 or 126 or 127 or 128 (41939)

130 4 and 129 (829)

131 limit 130 to clinical trial, all (15)

132 10 and 130 (62)

133 131 or 132 (68)

134 limit 133 to (english or german) (66)

135 limit 134 to dt=20180904-20230417 (33)

136 limit 134 to ed=20180904-20230417 (28)

137 135 or 136 (35)

138 polypectom*.mp. (5810)

139 proctectom*.mp. (2870)

140 rectopex*.mp. (1014)

141 ((rect* or colo?rect* or meso?rect* or polyp* or sphincter*) adj3 (remov* or excis* or resect*)).mp. (28659)

142 colo?rectom*.mp. (25)

143 rectom*.mp. (48)

144 138 or 139 or 140 or 141 or 142 or 143 (35415)

145 4 and 144 (1214)

146 limit 145 to clinical trial, all (35)

147 10 and 145 (149)

148 146 or 147 (163)

149 limit 148 to (english or german) (153)

150 limit 149 to dt=20180907-20230417 (78)

151 limit 149 to ed=20180907-20230417 (68)

152 150 or 151 (88)

153 ((gallbladder* or gall bladder*) adj3 (remov* or excis* or resect*)).mp. (2035)

154 exp Cholecystectomy/ (30690)

155 cholecystectom*.mp. (42635)

156 153 or 154 or 155 (43380)

157 4 and 156 (541)

158 limit 157 to clinical trial, all (27)

159 10 and 157 (49)

160 158 or 159 (55)

161 limit 160 to (english or german) (50)

162 limit 161 to dt=20180911-20230417 (16)

163 limit 161 to ed=20180911-20230417 (21)

164 162 or 163 (23)

165 exp Herniorrhaphy/ (11070)

166 herniorrhaph*.mp. (13072)

167 hernioplast*.mp. (1888)

168 (hernia* adj3 repair*).mp. (16769)

169 165 or 166 or 167 or 168 (24152)

170 4 and 169 (626)

171 limit 170 to clinical trial, all (18)

172 10 and 170 (37)

173 171 or 172 (41)

174 limit 173 to (english or german) (40)

175 limit 174 to dt=20180914-20230417 (32)

176 limit 174 to ed=20180914-20230417 (27)

177 175 or 176 (33)

178 remove duplicates from 177 (32)

179 ((liver* or hepat*) adj3 (remov* or excis* or resect*)).mp. (31575)

180 exp Hepatectomy/ (34106)

181 Hepatectom*.mp. (43721)

182 179 or 180 or 181 (58840)

183 4 and 182 (645)

184 limit 183 to clinical trial, all (4)

185 10 and 183 (27)

186 184 or 185 (31)

187 limit 186 to (english or german) (29)

188 limit 187 to dt=20180913-20230417 (20)

189 limit 187 to ed=20180913-20230417 (17)

190 188 or 189 (22)

191 16 or 31 or 51 or 65 or 82 or 95 or 110 or 121 or 137 or 152 or 164 or 178 or 190 (310)

***************************

17.04.2023

**Database: Ovid MEDLINE(R) ALL <1946 to April 17, 2023>**

Search Strategy:

--------------------------------------------------------------------------------

1 exp Robotic Surgical Procedures/ (15378)

2 robot*-assisted*.mp. (19699)

3 (robot* adj5 (surger* or surgical*)).mp. (27114)

4 1 or 2 or 3 (34456)

5 ((pulmonary or lung*) adj5 (segmentectom* or lobectom*)).mp. (5573)

6 ((excis* or resect*) adj5 (lobe* or lung*)).mp. (26295)

7 5 or 6 (30117)

8 4 and 7 (539)

9 limit 8 to clinical trial, all (11)

10 ((randomized controlled trial or controlled clinical trial).pt. or randomized.ab. or placebo.ab. or clinical trials as topic.sh. or randomly.ab. or trial.ti.) not (exp animals/ not humans.sh.) (1396593)

11 8 and 10 (30)

12 9 or 11 (34)

13 limit 12 to (english or german) (33)

14 limit 13 to dt=20180626-20230417 (19)

15 limit 13 to ed=20180626-20230417 (12)

16 14 or 15 (19)

17 exp Mediastinum/su [Surgery] (1016)

18 (mediastin* adj5 (surg* or resect*)).mp. (5255)

19 exp Thymectomy/ (8271)

20 thymectom*.mp. (11056)

21 exp Thymus Gland/su [Surgery] (517)

22 (thymus adj5 (surg* or resect* or excis* or remov*)).mp. (879)

23 17 or 18 or 19 or 20 or 21 or 22 (17420)

24 4 and 23 (383)

25 limit 24 to clinical trial, all (5)

26 10 and 24 (17)

27 25 or 26 (20)

28 limit 27 to (english or german) (19)

29 limit 28 to dt=20180704-20230417 (10)

30 limit 28 to ed=20180704-20230417 (8)

31 29 or 30 (11)

32 exp Gastroesophageal Reflux/ (29101)

33 reflux.mp. (69492)

34 GER.mp. (3724)

35 GERD.mp. (10372)

36 GORD.mp. (909)

37 (plication* or fundic wrap*).mp. (4336)

38 anti-reflux.mp. (1764)

39 anti?reflux.mp. (4842)

40 exp FUNDOPLICATION/ (5080)

41 fundoplication*.mp. (7847)

42 32 or 33 or 34 or 35 or 36 or 37 or 38 or 39 or 40 or 41 (78713)

43 4 and 42 (505)

44 limit 43 to clinical trial, all (18)

45 10 and 43 (34)

46 44 or 45 (41)

47 45 or 46 (41)

48 limit 47 to (english or german) (38)

49 limit 48 to dt=20180801-20230417 (7)

50 limit 48 to ed=20180801-20230417 (6)

51 49 or 50 (8)

52 exp Esophagectomy/ (12348)

53 Oesophagectom*.mp. (1886)

54 Esophagectom*.mp. (16996)

55 ((Trans?hiat* or Trans-hiat*) adj3 (Oesophagectom* or Esophagectom*)).mp. (885)

56 ((oesophag* or esophag*) adj3 (remov* or excis* or resect*)).mp. (7308)

57 52 or 53 or 54 or 55 or 56 (22007)

58 4 and 57 (516)

59 limit 58 to clinical trial, all (18)

60 10 and 58 (55)

61 59 or 60 (59)

62 limit 61 to (english or german) (56)

63 limit 62 to dt=20180808-20230417 (39)

64 limit 62 to ed=20180808-20230417 (31)

65 63 or 64 (40)

66 exp Esophageal Perforation/ (4518)

67 ((oesophag* or esophag* or Heller*) adj3 (repair* or perforat* or myotom*)).mp. (8833)

68 exp Heller Myotomy/ (266)

69 LHM.ti,ab. (383)

70 exp Esophageal Achalasia/ (7666)

71 achalasia*.mp. (9614)

72 ((oesophag* or esophag*) adj3 (swallow* adj3 (disorder* or difficult* or problem* or impair*))).mp. (73)

73 ((oesophag* or esophag*) adj3 dysphagia*).mp. (1632)

74 66 or 67 or 68 or 69 or 70 or 71 or 72 or 73 (19055)

75 4 and 74 (124)

76 limit 75 to clinical trial, all (4)

77 10 and 75 (20)

78 76 or 77 (23)

79 limit 78 to (english or german) (20)

80 limit 79 to dt=20180822-20230417 (6)

81 limit 79 to ed=20180822-20230417 (4)

82 80 or 81 (6)

83 exp Gastrectomy/ (40638)

84 Gastrectom*.mp. (53221)

85 Pylorectom*.mp. (72)

86 ((stomach or pylor*) adj3 (remov* or excis* or resect*)).mp. (3690)

87 83 or 84 or 85 or 86 (55432)

88 4 and 87 (744)

89 limit 88 to clinical trial, all (32)

90 10 and 88 (90)

91 89 or 90 (103)

92 limit 91 to (english or german) (100)

93 limit 92 to dt=20180824-20230417 (45)

94 limit 92 to ed=20180824-20230417 (44)

95 93 or 94 (48)

96 exp Bariatric Surgery/ (32991)

97 bariatric*.mp. (28864)

98 (Gastric adj3 (bypass* or band* or stimul*)).mp. (24237)

99 Roux*.mp. (17452)

100 RYGB.ti,ab. (4110)

101 (sleeve* adj3 gastrectom*).mp. (8475)

102 96 or 97 or 98 or 99 or 100 or 101 (56694)

103 4 and 102 (527)

104 limit 103 to clinical trial, all (8)

105 10 and 103 (32)

106 104 or 105 (35)

107 limit 106 to (english or german) (33)

108 limit 107 to dt=20180830-20230417 (13)

109 limit 107 to ed=20180830-20230417 (16)

110 108 or 109 (17)

111 exp Intestine, Small/ (167606)

112 ((small bowel* or small intestine*) adj3 (remov* or excis* or resect*)).mp. (4526)

113 111 or 112 (169731)

114 4 and 113 (230)

115 limit 114 to clinical trial, all (2)

116 10 and 114 (7)

117 115 or 116 (9)

118 limit 117 to (english or german) (8)

119 limit 118 to dt=20180831-20230417 (2)

120 limit 118 to ed=20180831-20230417 (4)

121 119 or 120 (4)

122 exp Colectomy/ (23297)

123 colectom*.mp. (26875)

124 procto?colectom*.mp. (5407)

125 hemi?colectom*.mp. (5017)

126 sigmoidectom*.mp. (1226)

127 transversectom*.mp. (31)

128 ((colon* or hemi*colon* or sigmoid*) adj3 (remov* or excis* or resect*)).mp. (11060)

129 122 or 123 or 124 or 125 or 126 or 127 or 128 (41936)

130 4 and 129 (829)

131 limit 130 to clinical trial, all (15)

132 10 and 130 (62)

133 131 or 132 (68)

134 limit 133 to (english or german) (66)

135 limit 134 to dt=20180904-20230417 (33)

136 limit 134 to ed=20180904-20230417 (28)

137 135 or 136 (35)

138 polypectom*.mp. (5810)

139 proctectom*.mp. (2868)

140 rectopex*.mp. (1014)

141 ((rect* or colo?rect* or meso?rect* or polyp* or sphincter*) adj3 (remov* or excis* or resect*)).mp. (28656)

142 colo?rectom*.mp. (25)

143 rectom*.mp. (48)

144 138 or 139 or 140 or 141 or 142 or 143 (35411)

145 4 and 144 (1214)

146 limit 145 to clinical trial, all (35)

147 10 and 145 (149)

148 146 or 147 (163)

149 limit 148 to (english or german) (153)

150 limit 149 to dt=20180907-20230417 (78)

151 limit 149 to ed=20180907-20230417 (68)

152 150 or 151 (88)

153 ((gallbladder* or gall bladder*) adj3 (remov* or excis* or resect*)).mp. (2035)

154 exp Cholecystectomy/ (30688)

155 cholecystectom*.mp. (42633)

156 153 or 154 or 155 (43378)

157 4 and 156 (541)

158 limit 157 to clinical trial, all (27)

159 10 and 157 (49)

160 158 or 159 (55)

161 limit 160 to (english or german) (50)

162 limit 161 to dt=20180911-20230417 (16)

163 limit 161 to ed=20180911-20230417 (21)

164 162 or 163 (23)

165 exp Herniorrhaphy/ (11068)

166 herniorrhaph*.mp. (13070)

167 hernioplast*.mp. (1888)

168 (hernia* adj3 repair*).mp. (16766)

169 165 or 166 or 167 or 168 (24148)

170 4 and 169 (626)

171 limit 170 to clinical trial, all (18)

172 10 and 170 (37)

173 171 or 172 (41)

174 limit 173 to (english or german) (40)

175 limit 174 to dt=20180914-20230417 (32)

176 limit 174 to ed=20180914-20230417 (27)

177 175 or 176 (33)

178 remove duplicates from 177 (32)

179 ((liver* or hepat*) adj3 (remov* or excis* or resect*)).mp. (31570)

180 exp Hepatectomy/ (34101)

181 Hepatectom*.mp. (43715)

182 179 or 180 or 181 (58833)

183 4 and 182 (644)

184 limit 183 to clinical trial, all (4)

185 10 and 183 (27)

186 184 or 185 (31)

187 limit 186 to (english or german) (29)

188 limit 187 to dt=20180913-20230417 (20)

189 limit 187 to ed=20180913-20230417 (17)

190 188 or 189 (22)

***************************

17.04.2023

**Search Name: Robotic Surgery (Update 2023) Gesamt**

Last Saved: 19/04/2023 17:27:20

Comment: LG 19.04.2023

ID Search

#1 MeSH descriptor: [Robotic Surgical Procedures] explode all trees

#2 (robot* assisted*) (Word variations have been searched)

#3 robot* near surg* (Word variations have been searched)

#4 (#1 OR #2 OR #3)

#5 (pulmonar* or lung*) near (segmentectom* or lobectom*) (Word variations have been searched)

#6 (excis* or resect*) near (lobe* or lung*) (Word variations have been searched)

#7 #5 or #6

#8 #4 AND #7 with Cochrane Library publication date Between Jun 2018 and Apr 2023, in Trials

#9 (conference proceeding):pt

#10 (abstract):so

#11 (clinicaltrials OR trialsearch OR ANZCTR OR ensaiosclinicos OR Actrn OR chictr OR cris OR ctri OR registroclinico OR clinicaltrialsregister OR DRKS OR IRCT OR Isrctn OR rctportal OR JapicCTI OR JMACCT OR jRCT OR JPRN OR Nct OR UMIN OR trialregister OR PACTR OR R.B.R.OR REPEC OR SLCTR OR Tcr):so (Word variations have been searched)

#12 #9 OR #10 OR #11

#13 #8 NOT #12

#14 MeSH descriptor: [Mediastinum] explode all trees

#15 mediastin* near (surg* or resect*) (Word variations have been searched)

#16 MeSH descriptor: [Thymectomy] explode all trees

#17 thymectom* (Word variations have been searched)

#18 MeSH descriptor: [Thymus Gland] explode all trees

#19 thymus near (surg* or resect* or excis* or remov*) (Word variations have been searched)

#20 #14 or #15 or #16 or #17 or #18 or #19 (Word variations have been searched)

#21 #4 and #20 with Cochrane Library publication date Between Jul 2018 and Apr 2023, in Trials

#22 #21 NOT #12

#23 MeSH descriptor: [Gastroesophageal Reflux] explode all trees

#24 gastro*esophageal reflux (Word variations have been searched)

#25 GER:ti,ab,kw

#26 GERD:ti,ab,kw

#27 GORD:ti,ab,kw

#28 (anti*reflux or reflux) near (surg* or operat* or management) (Word variations have been searched)

#29 MeSH descriptor: [Fundoplication] explode all trees

#30 fundoplication* (Word variations have been searched)

#31 plication* or fundic wrap* (Word variations have been searched)

#32 #23 or #24 or #25 or #26 or #27 #28 or #29 or #30 or #31

#33 #4 and #32 with Cochrane Library publication date Between Aug 2018 and Apr 2023, in Trials

#34 #33 NOT #12

#35 MeSH descriptor: [Esophagectomy] explode all trees

#36 Oesophagectom* (Word variations have been searched)

#37 Esophagectom* (Word variations have been searched)

#38 (Transhiat* OR Trans-hiat*) NEAR (Oesophagectom* OR Esophagectom*) (Word variations have been searched)

#39 (oesophag* OR esophag*) NEAR (remov* OR excis* OR resect*) (Word variations have been searched)

#40 #35 OR #36 OR #37 OR #38 OR #39 (Word variations have been searched)

#41 #4 AND #40 with Cochrane Library publication date Between Aug 2018 and Apr 2023, in Trials

#42 #41 NOT #12

#43 MeSH descriptor: [Esophageal Perforation] explode all trees

#44 ((oesophag* OR esophag* OR Heller*) NEAR (repair* OR perforat* OR myotom*)) (Word variations have been searched)

#45 MeSH descriptor: [Heller Myotomy] explode all trees

#46 (LHM):ti,ab,kw

#47 MeSH descriptor: [Esophageal Achalasia] explode all trees

#48 (achalasia*) (Word variations have been searched)

#49 ((oesophag* OR esophag*) NEAR (swallow* NEAR (disorder* OR difficult* OR problem* OR impair*))) (Word variations have been searched)

#50 ((oesophag* OR esophag*) NEAR dysphagia*) (Word variations have been searched)

#51 #43 OR #44 OR #45 OR #46 OR #47 OR #48 OR #49 OR #50

#52 #4 AND #51 with Cochrane Library publication date Between Aug 2018 and Apr 2023, in Trials

#53 #52 NOT #12

#54 MeSH descriptor: [Gastrectomy] explode all trees

#55 (Gastrectom*) (Word variations have been searched)

#56 (Pylorectom*) (Word variations have been searched)

#57 (stomach OR pylor*) NEAR (remov* OR excis* OR resect*)

#58 #54 OR #55 OR #56 OR #57 (Word variations have been searched)

#59 #4 AND #58 with Cochrane Library publication date Between Aug 2018 and Apr 2023, in Trials

#60 #59 NOT #12

#61 MeSH descriptor: [Bariatric Surgery] explode all trees

#62 (bariatric*) (Word variations have been searched)

#63 ((Gastric*) NEAR (bypass* OR band* OR stimul*)) (Word variations have been searched)

#64 (Roux*) (Word variations have been searched)

#65 (RYGB):ti,ab,kw

#66 (sleeve* NEAR gastrect*) (Word variations have been searched)

#67 #61 OR #62 OR #63 OR #64 OR #65 OR #66

#68 #4 AND #67 with Cochrane Library publication date Between Aug 2018 and Apr 2023, in Trials

#69 #68 NOT #12

#70 MeSH descriptor: [Intestine, Small] explode all trees

#71 (small bowel* OR small intestine*) NEAR (remov* OR excis* OR resect*) (Word variations have been searched)

#72 #70 OR #71

#73 #4 AND #72 with Cochrane Library publication date Between Sep 2018 and Apr 2023, in Trials

#74 #73 NOT #12

#75 MeSH descriptor: [Colectomy] explode all trees

#76 colectom* (Word variations have been searched)

#77 procto*colectom* (Word variations have been searched)

#78 hemi*colectom* (Word variations have been searched)

#79 sigmoidectom* (Word variations have been searched)

#80 transversectom* (Word variations have been searched)

#81 (colon* OR hemi*colon* OR sigmoid*) NEAR (remov* OR excis* OR resect*) (Word variations have been searched)

#82 #75 OR #76 OR #77 OR #78 OR #79 OR #80 OR #81 (Word variations have been searched)

#83 #4 AND #82 with Cochrane Library publication date Between Sep 2018 and Apr 2023, in Trials

#84 #83 NOT #12

#85 colo*rectom* (Word variations have been searched)

#86 rectom* (Word variations have been searched)

#87 polypectom* (Word variations have been searched)

#88 proctectom* (Word variations have been searched)

#89 rectopex* (Word variations have been searched)

#90 (rect* OR colo*rect* OR meso*rect* OR polyp* OR sphincter*) NEAR (remov* OR excis* OR resect*) (Word variations have been searched)

#91 #85 OR #86 OR #87 OR #88 OR #89 OR #90 (Word variations have been searched)

#92 #4 AND #91 with Cochrane Library publication date Between Sep 2018 and Apr 2023, in Trials

#93 #92 NOT #12

#94 MeSH descriptor: [Cholecystectomy] explode all trees

#95 Cholecystectom* (Word variations have been searched)

#96 (gallbladder* OR gall bladder*) NEAR (remov* OR excis* OR resect*) (Word variations have been searched)

#97 #94 OR #95 OR #96 (Word variations have been searched)

#98 #4 AND #97 with Cochrane Library publication date Between Sep 2018 and Apr 2023, in Trials

#99 #98 NOT #12

#100 MeSH descriptor: [Herniorrhaphy] 1 tree(s) exploded

#101 Herniorrhaph* (Word variations have been searched)

#102 Hernioplast* (Word variations have been searched)

#103 hernia* NEAR repair* (Word variations have been searched)

#104 #100 OR #101 OR #102 OR #103 (Word variations have been searched)

#105 #4 AND #104 with Cochrane Library publication date Between Sep 2018 and Apr 2023, in Trials

#106 #105 NOT #12

#107 (liver* OR hepat*) NEAR (remov* OR excis* OR resect*) (Word variations have been searched)

#108 MeSH descriptor: [Hepatectomy] explode all trees

#109 Hepatectom* (Word variations have been searched)

#110 #107 OR #108 OR #109 (Word variations have been searched)

#111 #4 AND #110 with Cochrane Library publication date Between Sep 2018 and Apr 2023, in Trials

#112 #111 NOT #12

#113 #13 OR #22 OR #34 OR #42 OR #53 OR #60 OR #69 OR #74 OR #84 OR #93 OR #99 OR #106 OR #112

174 Hits
